# The Circadian Transcription Factor CLOCK Modulates Oxidative Stress Resistance via the ACHL–*Relish* Axis in Drosophila

**DOI:** 10.1002/advs.202514388

**Published:** 2025-10-30

**Authors:** Xu Liu, Jiajia Fang, Dawei Cheng, Wei Luan, Yan Lv, Wen Hu, Lei Pan, Yong Zhang

**Affiliations:** ^1^ Jiangsu Key Laboratory of Drug Discovery and Translational Research for Brain Diseases Cambridge‐Suda Genomic Resource Center The Fourth Affiliated Hospital, Suzhou Medical College Soochow University Suzhou Jiangsu 215123 China; ^2^ State Key Laboratory of Immune Response and Immunotherapy Shanghai Institute of Immunity and Infection Chinese Academy of Sciences Shanghai 200031 China

**Keywords:** anti‐microbial peptides, circadian rhythm, CLOCK, oxidative stress, Relish/NF‐κB signaling, RNA‐binding protein ACHL

## Abstract

Circadian clocks orchestrate temporal regulation of diverse physiological processes, including innate immunity and oxidative stress responses. However, the molecular mechanisms by which core clock components modulate immune tone and redox homeostasis remain elusive. Here, the circadian transcription factor CLOCK (CLK) is identified as a key regulator of oxidative stress resistance in *Drosophila melanogaster*. Loss of *clk* significantly enhances survival under oxidative stress, accompanied by constitutive activation of innate immune pathways. Mechanistically, the RNA‐binding protein Achilles (ACHL) is identified as a critical downstream effector of CLK. Indeed, CLK drives rhythmic transcription of *Achl*, and ACHL post‐transcriptionally represses the NF‐κB homolog Relish by promoting its mRNA degradation, thereby limiting immune overactivation. Disruption of this regulatory cascade, through loss of either *clk* or *Achl*, leads to increased Relish abundance, excessive immune gene expression, and enhanced oxidative stress resistance. Genetic suppression of *Relish* reverses these phenotypes, establishing a functional CLK–ACHL*–Relish* axis that links circadian output to immune restraint. Importantly, this regulatory mechanism is evolutionarily conserved, as *Clock*‐deficient mammalian cells exhibit increased resistance to oxidative injury. Together, the findings uncover a post‐transcriptional immune checkpoint controlled by circadian networks, linking immune quiescence with redox adaptation.

## Graphical abstract

This study identifies the circadian transcription factor CLOCK (CLK) as a key regulator of oxidative stress resistance in fruit flies. CLK controls immune responses by driving rhythmic transcription of an RNA‐binding protein‐Achilles (ACHL) in the fly brain, which post‐transcriptionally represses the NF‐κB homolog Relish, limiting immune activation and promoting redox homeostasis.

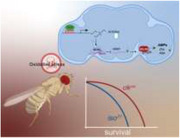

## 1 Introduction

Circadian rhythms are intrinsic ≈24‐h oscillations that temporally coordinate a wide range of physiological and behavioral processes, including sleep–wake cycles, metabolism, immune responses, and stress adaptation.^[^ ^1^, ^2^, ^3^, ^4^ ^]^ In *Drosophila melanogaster*, circadian rhythms are driven by a conserved transcription–translation feedback loop (TTFL) involving core pacemaker genes such as *clock (clk)*, *cycle (cyc)*, *period (per*), and *timeless (tim)*. The transcription factors CLK and CYC form a heterodimer that binds E‐box motifs to activate *per and tim* transcription, while PER and TIM feedback to repress CLK–CYC activity.^[^ ^5^, ^6^, ^7^, ^8^ ^]^ The circadian locomotor rhythm of *Drosophila* is driven by ≈150 circadian neurons in the brain, which are labeled by pacemaker proteins, such as PER. These circadian neurons are anatomically classified into six groups, including the small and large ventral lateral neurons (s‐LNvs, l‐LNvs, including the fifth s‐LNv), dorsal lateral neurons (LNds), and three subtypes of dorsal neurons (DN1s, DN2s, and DN3s). Among these, the PDF‐expressing s‐LNvs act as master pacemakers that sustain circadian behavioral rhythms under constant darkness.^[^ ^9^, ^10^, ^11^ ^]^ The molecular oscillator functions not only in the pacemaker neurons of the fly brain, but also in peripheral tissues, ensuring systemic circadian coordination.^[^ ^11^ ^]^

Perturbation of circadian homeostasis‐whether due to genetic mutation, environmental desynchrony, or aging‐has been implicated in the pathogenesis of various disorders, including chronic inflammation, metabolic dysregulation, neurodegeneration, and cancer.^[^ ^12^, ^13^, ^14^, ^15^ ^]^ In mammals, circadian transcription factors such as CLOCK and BMAL1 modulate inflammatory responses through crosstalk with NF‐κB, MAPK, and glucocorticoid signaling pathways.^[^ ^16^, ^17^, ^18^ ^]^ Notably, the link between circadian regulation and immune function is evolutionarily conserved.^[^ ^19^ ^]^ In *Drosophila*, *clock* mutations disrupt the expression of antimicrobial peptide (AMP) genes, underscoring a potential link between circadian rhythms and innate immunity.^[^ ^20^ ^]^ Furthermore, basal AMP transcript levels display time‐of‐day oscillations, and TIM further modulates phagocytic activity, driving daily fluctuations in antibacterial defense.^[^ ^21^ ^]^ Moreover, survival following bacterial infection displays circadian gating, with peak resistance observed at night, which is abolished in *per^01^*, *Clk^Jrk^*, and *tim^01^* mutants. Interestingly, while *per^01^* flies show reduced survival, *Clk^Jrk^* mutant exhibits enhanced resistance. This observation indicates gene‐specific roles of circadian regulators in modulating host immunity, with the underlying mechanisms remaining unknown.^[^ ^22^ ^]^ AMPs are evolutionarily conserved cationic molecules that serve as frontline effectors of innate immunity by disrupting microbial membranes.^[^ ^23^ ^]^ In *Drosophila*, AMP genes such as *Metchnikowin (Mtk)*,^[^ ^24^ ^]^ *Drosocin (Dro)*,^[^ ^25^ ^]^ *Drosomycin (Drs)*,^[^ ^26^ ^]^ and *Attacin (AttA)* ^[^ ^27^ ^]^ are robustly induced in the fat body upon systemic infection, while many also exhibit tissue‐specific expression in the gut, trachea, nervous system, and reproductive organs.^[^ ^28^, ^29^ ^]^ Their transcription is orchestrated by the Toll and IMD signaling pathways via NF‐κB family factors, rendering AMP mRNA levels reliable readouts of immune pathway activation .^[^ ^30^ ^]^

Oxidative stress, a common physiological challenge associated with infection, inflammation, environmental toxins, and aging, arises from the accumulation of reactive oxygen species (ROS) and disruption of redox homeostasis.^[^ ^31^, ^32^, ^33^, ^34^ ^]^ Circadian regulators have been implicated in antioxidant defense, for instance, *Bmal1*‐deficient mice exhibit premature aging and elevated oxidative burden,^[^ ^35^ ^]^ while clock mutants in *Drosophila* show altered expression of detoxifying enzymes such as *catalase* and *gstD1*.^[^ ^36^ ^]^ However, how the circadian clocks interact with immune signaling, especially under stress conditions, remain unexplored.

In this study, we identify *Achilles (Achl)*,^[^ ^37^ ^]^ a putative neuronal specific RNA binding protein (RBP), as a direct target of CLK that functions to repress the NF‐κB homolog *Relish* (*Rel*) post‐transcriptionally. We demonstrate that CLK activates *Achl* transcription via conserved E‐box elements in the promoter, and that ACHL binds *Rel* mRNA to promote its degradation, thereby limiting AMPs production and maintaining immune quiescence. Loss of either *clk* or *Achl* elevates Rel abundance, which correlates with upregulation of AMP genes and increased survival under oxidative stress. This phenotype reflects immune reprogramming accompanied by compensatory antioxidant enzyme activity. Furthermore, *Clock*‐deficient NIH‐3T3 cells exhibit reduced H_2_O_2_‐induced cytotoxicity, suggesting that the circadian control of immune–redox balance is conserved across species.

Taken together, our findings uncover a gated CLK–ACHL*–Relish* axis that integrates transcriptional and post‐transcriptional control to coordinate immune homeostasis and oxidative stress adaptation. This work provides mechanistic insight into how circadian disruption reshapes immune tone and redox resilience, with broader implications for the management of inflammation, aging, and oxidative stress‐related pathologies.

## 2 Result

### 2.1 Loss of CLOCK Confers Oxidative Stress Resistance in *Drosophila*

**Figure 1:**
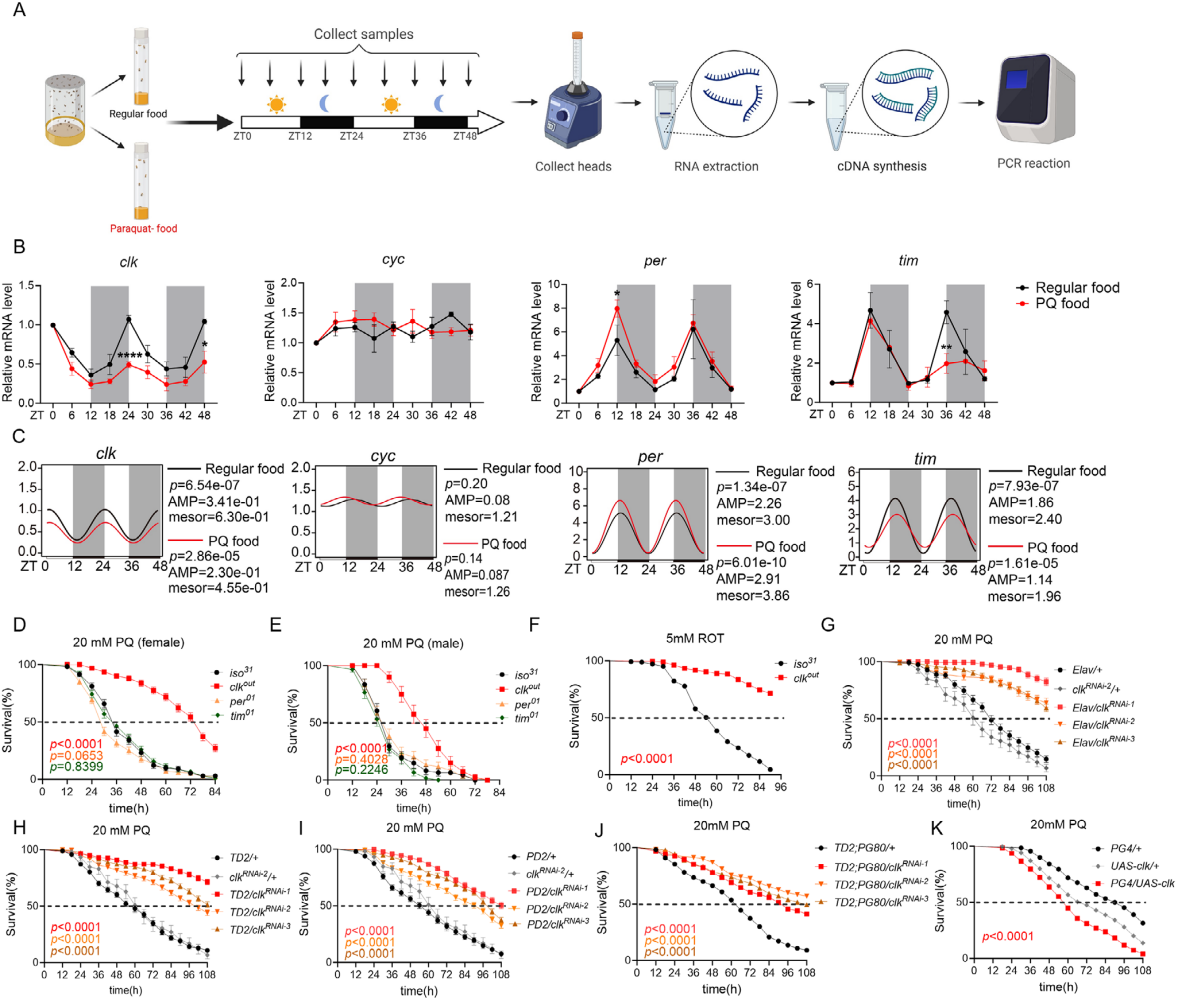
*clk* mutants exhibited increased survival under paraquat‐induced oxidative stress in *Drosophila*. A) Schematic of experimental design. Flies were maintained under a 12:12 h light–dark (LD) cycle on standard or 20 mm paraquat (PQ)‐supplemented food. Samples were collected every 6 h over a 48‐h period at designated Zeitgeber times (ZTs). B) Temporal expression profiles of core clock genes (*clk*, *cyc, per*, *tim*) in wild‐type (*iso^31^*) flies under control (black) and PQ‐treated (red) conditions. mRNA levels were measured by qPCR. Gray shading indicates the dark phase (ZT12–24). Statistical significance was assessed using a two‐tailed unpaired *t*‐test ^(*^ *p* < 0.05, ^**^ *p* < 0.01, ^****^ *p* < 0.0001). Data are from three biological repeats. C) Cosinor–circacompare analysis of rhythmicity parameters, including mesor (mean expression), amplitude, and phase. Expression curves depict modeled oscillatory profiles for each gene. D,E) Survival analysis of female (D) and male (E) flies under PQ exposure. *clk^out^* mutants displayed significantly increased survival compared to *iso^31^* controls. Additional circadian mutants (*per^01^*, *tim^01^*) were included for comparison. Survival curves were analyzed by the log‐rank test. F) Survival analysis of female *clk^out^* and control flies under oxidative stress induced by 5 mm rotenone. G–I) Neuron‐specific knockdown of *clk* enhances oxidative stress resistance. RNAi targeting of *clk* in pan‐neuronal (*Elav‐GAL4*, F), circadian cells (*tim‐GAL4; UAS‐Dicer2*, G), and PDF‐positive neurons (*PD2* = *PDF‐GAL4; UAS‐Dicer2*, H) improved survival under PQ treatment. J) Use of PDF‐GAL80 to restrict *clk* knockdown to PDF‐negative neurons partially suppressed the protective phenotype. K) Overexpression of *clk* in PDF‐positive neurons (*PG4 = PDF‐GAL4*) reduced survival upon PQ challenge. All survival experiments were performed with at least three independent biological replicates. Data represent mean ± SEM. Statistical comparisons were conducted using the log‐rank test.

The circadian clock plays a fundamental role in maintaining physiological homeostasis, yet its contribution to oxidative stress adaptation remains incompletely understood. To explore the effects of oxidative stress on circadian gene expression, we first exposed flies to paraquat (PQ)‐containing food to induce oxidative stress under 12:12 h light–dark (LD) conditions, and collected fly head samples every 6 h for 48 h (**Figure** **1**A). Indeed, PQ treatment markedly altered both the amplitude and overall expression levels of core pacemaker genes (*clk*, *cyc, per*, and *tim*), with minimal impact on their circadian phase compared with flies maintained on a standard diet (Figure 1B). Among these, *clk* exhibited the most pronounced changes compared to other genes (Figure 1B). Cosinor‐circacompare analysis^[^ ^38^ ^]^ further confirmed significant changes in mesor, amplitude, and phase parameters (Figure 1C), indicating that oxidative stress disrupts the endogenous circadian pacemaker genes expression. We next assessed the physiological relevance of these changes using circadian gene mutants (*clk^out^*, *per^01^*, *tim^01^*) upon PQ exposure at ZT (zeitegeber time) 1 (1 h after light on). While functional interrogation of other circadian mutants revealed no obvious phenotype, notably, *clk^out^* flies of both sexes exhibited significantly prolonged survival under 20 mm PQ compared to *iso^31^* controls (Figure 1D,E). In contrast, *clk^out^* flies exhibited a reduced lifespan when maintained on regular food (Figure S1A, Supporting Information). This survival advantage persisted at higher PQ concentrations and was recapitulated under another oxidation inducer‐rotenone induced stress (Figure 1F; Figure S1B,C, Supporting Information), highlighting a specific role for *clk* in modulating oxidative resilience. Based on these findings, *clk* was selected for further mechanistic investigation. Surprisingly, despite improved survival, *clk^out^* flies accumulated elevated levels of reactive oxygen species (ROS) in the brain, as revealed by DCFH‐DA staining (Figure S1D, Supporting Information), suggesting that enhanced oxidative resistance stems from increased ROS tolerance rather than decreased ROS production.

Next, we further dissected the cellular basis of this phenotype by selectively knocking down *clk* using GAL4/UAS system. Pan‐neuronally downregulation of *clk* with different RNAi lines using *Elav‐GAL4* indeed phenocopied the effects of *clk^out^* mutants, suggesting that *clk* is mainly required in neurons for the oxidative resistance. Specifically, we further restricted *clk* downregulation in the circadian cells with *TD2* (*tim‐GAL4, UAS‐dicer2*), and observed a similar phenotype with pan‐neuronal knockdown, suggesting that *clk* is mainly required in circadian neurons for this phenotype. Consistent with this, *clk* knockdown within master pacemaker neurons using *PDF‐GAL4* also significantly extended fly survival upon PQ exposure (Figure 1G,I; Figure S1E, Supporting Information). A comparable phenotype was observed following *cyc* knockdown in the same neuronal populations (Figure S1F,H, Supporting Information), implicating the CLK–CYC transcriptional complex as a key mediator of oxidative stress susceptibility. Notably, restricting knockdown to PDF‐negative circadian neurons using *PDF‐GAL80* partially attenuated the survival benefit (Figure 1J). In contrast with the downregulation, overexpression of *clk* in PDF‐positive neurons reduced fly survival (Figure 1K), supporting a detrimental role for neuronal CLK during oxidative challenge. Lastly, targeted knockdown of *clk* or *cyc* in noncircadian mushroom body neurons using *MB‐GAL4* failed to confer any survival benefit (Figure S1I,J, Supporting Information), suggesting that the oxidative stress resistance phenotype is mediated by specific subsets of circadian neurons.

To exclude the possibility that the survival difference is due to altered PQ intake, feeding behavior was evaluated using the CAFE assay. In fact, *clk^out^* flies consumed significantly more PQ food over 24 and 48 h compared to *iso^31^* controls (Figure S1K,N, Supporting Information), indicating that increased survival is likely attributable to intrinsic physiological mechanisms rather than reduced intake or toxin avoidance.

Together, these findings position CLK as a critical regulator of organismal vulnerability to oxidative damage. Depletion of CLK—particularly within PDF‐positive pacemaker neurons—enhances survival under oxidative stress, highlighting a defined neural axis through which circadian transcription factors modulate stress resilience.

### 2.2 *clk* Deficiency Primes Basal Immune Activation and Modulates Stress Response Outcomes

**Figure 2:**
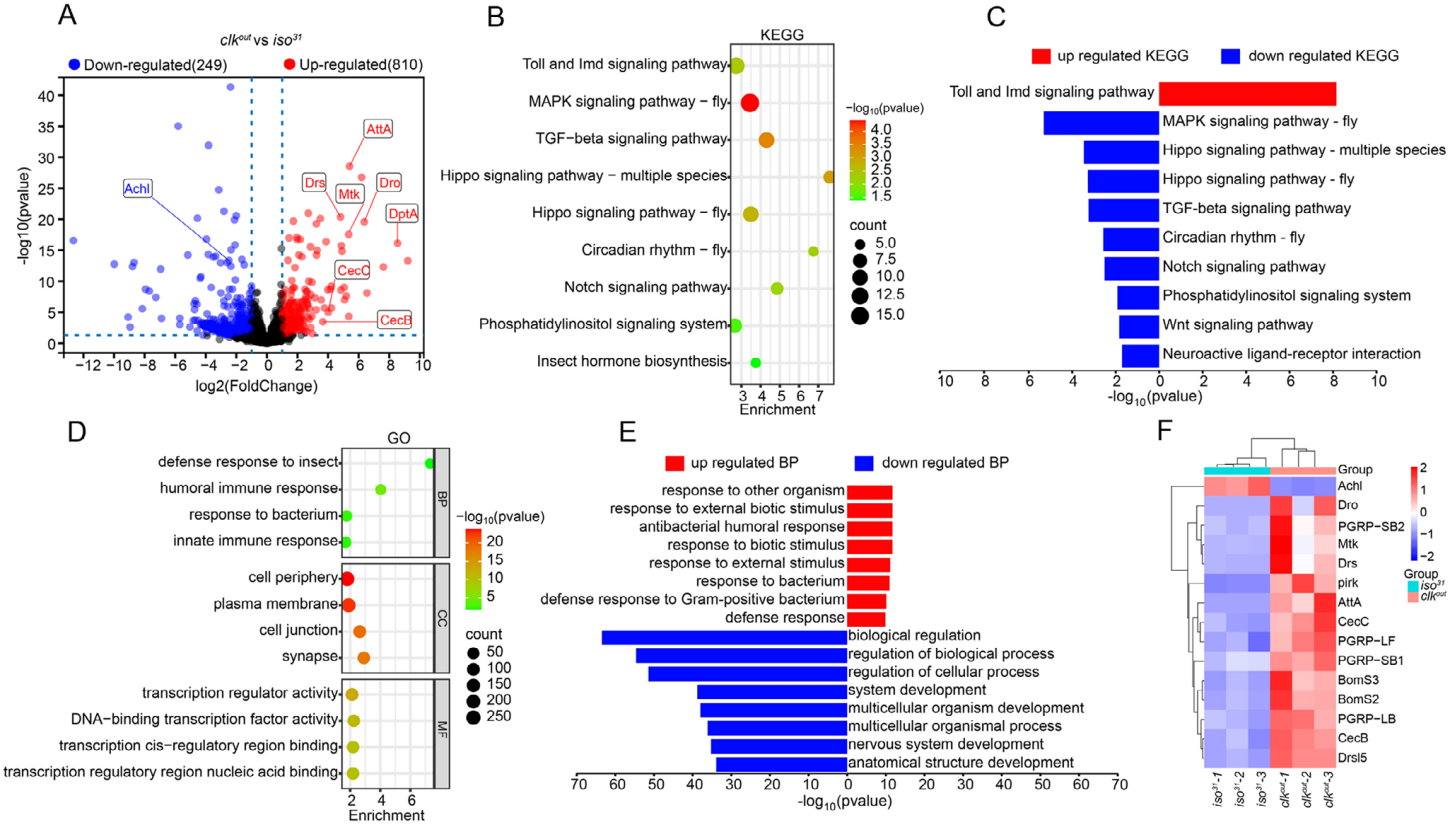
CLK modulates immune‐related gene expression and signaling pathway activity. A) Volcano plot showing differentially expressed genes (DEGs) in *clk^out^* mutants compared to *iso^31^* flies. Significantly upregulated (red) and downregulated (blue) genes were defined by |fold change| > 2 and *p* < 0.05. B) KEGG pathway enrichment analysis of DEGs in *clk^out^* flies. Immune‐related pathways, including Toll and IMD signaling, are prominently enriched. Dot size indicates the number of genes per pathway; color intensity reflects enrichment significance (–log_10_ P‐value). C) Comparative KEGG pathway enrichment for upregulated (red) and downregulated (blue) genes, highlighting immune activation and suppression of metabolic pathways. D) Gene Ontology (GO) enrichment analysis of DEGs across three major categories: biological process (BP), cellular component (CC), and molecular function (MF). E) GO biological process enrichment of upregulated (red) and downregulated (blue) genes in *clk^out^* flies. Upregulated genes are enriched in immune responses, including “response to bacterium” and “antibacterial humoral response.” F) Heat map of representative immune‐related and oxidative stress–associated genes in *clk^out^* versus *iso^31^* flies. Expression data represent three independent biological replicates.

Building upon the observation that *clk^out^* mutants exhibit enhanced resilience to oxidative stress, we conducted transcriptomic profiling of fly head to uncover the molecular basis of this phenotype. Differential expression analysis identified 345 upregulated and 353 downregulated genes in *clk^out^* flies relative to *iso^31^* controls (**Figure** **2**A). Kyoto Encyclopedia of Genes and Genomes (KEGG) enrichment of total differentially expressed genes (DEGs) identified innate immune pathways, including Toll and IMD signaling, as top‐ranked categories (Figure 2B). Stratifying DEGs into up‐ and down‐regulated subsets further refined this landscape: genes upregulated in *clk^out^* were significantly enriched for immune‐related pathways, whereas downregulated genes were primarily associated with the MAPK signaling pathway (Figure 2C). These findings suggest a global transcriptomic shift favoring immune activation–related signaling pathways in *clk^out^* mutants. This transcriptional reprogramming was further supported by Gene Ontology (GO) enrichment, which highlighted biological processes such as “defense response” and “response to bacterium” (Figure 2D). GO biological process (BP) enrichment of upregulated genes further emphasized pathways involved in innate immunity, including “response to bacterium” and “defense response to Gram‐positive bacterium” (Figure 2E). In line with these findings, heatmap analysis demonstrated increased basal expression of AMP‐related genes, including *Mtk*, *Drs*, and *AttA*, in *clk^out^* flies (Figure 2F), indicating that CLK functions to suppress immune gene expression. Together, these results collectively indicate that CLK plays a critical repressive role in regulating innate immune gene expression under normal conditions.

To determine whether this immune activation persists during prolonged oxidative challenge, another RNA‐seq analysis was conducted after 24 h of continuous PQ exposure. Similarly, volcano plot analysis again revealed significant upregulation of AMP‐related genes in *clk^out^* flies (Figure S2A, Supporting Information). Consistently, GO and KEGG enrichment analyses identified persistent activation of innate immune pathways, including Toll and IMD signaling (Figure S2B, Supporting Information), suggesting a prolonged immuno‐transcriptomic reprogramming in the absence of *clk* and upon PQ exposure.

To evaluate the functional consequences of immune priming, we first subjected flies to microbial challenges to activate the immune response and then put them under oxidative stress. Interestingly, heat‐inactivated *E. coli* injection prior to PQ exposure significantly improved survival in both wild‐type and *clk* mutants compared to PBS‐injected controls, while *clk^out^* flies consistently exhibiting superior resistance relative to *iso^31^* (Figure S2C, Supporting Information). These findings suggest that *clk* deficiency enhances the ability to tolerate combinatorial stress, consistent with the notion that a prior stimulation of immune response confers protection against subsequent oxidative stress. Supporting this, qRT‐PCR analysis revealed *E. coli*‐induced expression of AMP gene *AttA* in both genotypes, albeit with differing magnitudes and temporal patterns (Figure S2D, Supporting Information), pointing to altered immune dynamics in the absence of *clk*.

In addition to immune modulation, *clk* loss influenced redox homeostasis. Time‐course qPCR analyses showed that transcript levels of key antioxidant enzymes (*sod1*, *cat*, *gstd1*) were comparable between *clk^out^* and control, suggesting that CLK has a minimal effect on the antioxidant gene expression under oxidative stress treatment (Figure S3A, Supporting Information). However, catalase (CAT) enzymatic activity was consistently elevated in *clk^out^* flies across multiple time points (Figure S3B, Supporting Information), reflecting enhanced functional oxidative defense. Intriguingly, baseline hydrogen peroxide (H_2_O_2_) concentrations were significantly higher in *clk^out^* flies than *iso^31^* (Figure S3C, Supporting Information), suggesting an elevated oxidative burden that may act as a compensatory cue to activate enzymatic antioxidant responses. Together, these results indicate that CLK coordinates both immune and redox networks to determine systemic stress susceptibility.

Collectively, these findings demonstrate that *clk* deficiency induces constitutive activation of innate immune pathways—particularly AMP production—thereby reshaping the immune landscape and enhancing resilience to oxidative and immunological insults.

### 2.3 *Achl* Functions Downstream of CLK to Regulate Immune Homeostasis and Stress Resilience

**Figure 3:**
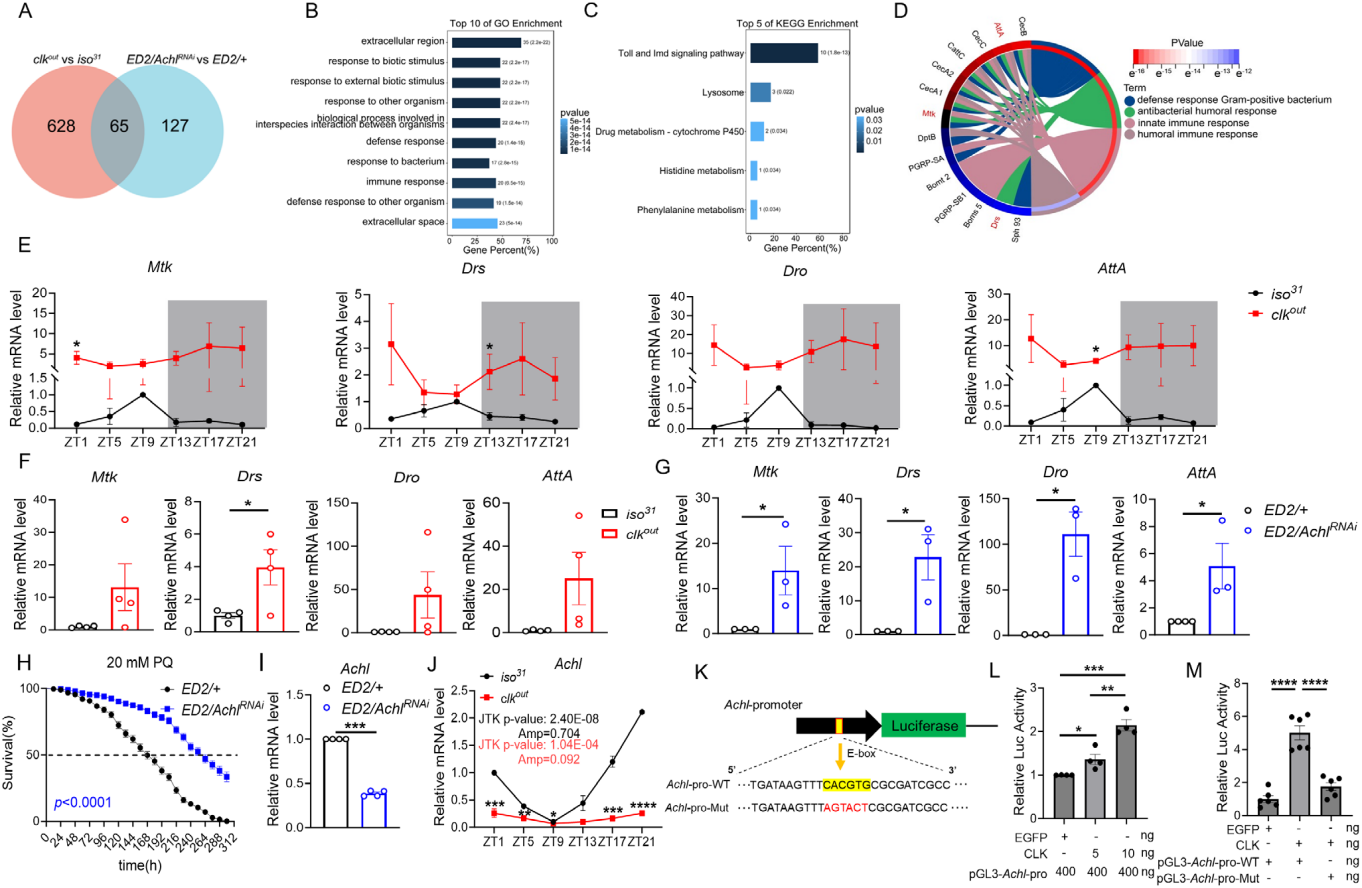
CLK regulates oxidative stress‐induced immune responses via *Achl*. A) Venn diagram showing 65 overlapping differentially expressed genes (DEGs) between *clk^out^* and *Elav‐GAL4*;; *UAS‐dicer2/Achl^RNAi^* flies, suggesting shared downstream pathways. B,C) GO B) and KEGG C) enrichment analyses of the shared DEGs, revealing enrichment in Toll/IMD immune signaling pathways. D) Chord diagram illustrating the association between key immune‐related DEGs and immune response categories, including defense against Gram‐positive bacteria, antibacterial humoral response, innate immunity, and humoral immunity. Color gradient indicates term significance (*p* < 10^−16^ to 10^−1^ ^2^). E) Temporal expression profiles of selected immune genes in *iso^31^* and *clk^out^* flies across circadian time points. Data are shown as mean ± SEM from four independent experiments, ^*^ *p* < 0.05 by two‐tailed unpaired *t*‐test. Data are from four biological repeats. F) Average AMP mRNA levels in *clk^out^* versus *iso^31^* flies measured by qRT‐PCR. Immune gene expression was significantly elevated in *clk^out^* flies (^*^ *p* < 0.05). Data are from four biological repeats. G) Relative mRNA levels of AMP genes in *Elav‐GAL4*; *UAS‐dicer2/Achl^RNAi^* versus control flies. Knockdown of *Achl* led to a significant increase in immune gene expression. ^*^ *p* < 0.05 by two‐tailed unpaired *t*‐test. Data are from three biological repeats. H) Survival analysis following pan‐neuronal *Achl* knockdown under paraquat exposure. *Elav‐GAL4*; *UAS‐dicer2/Achl^RNAi^* flies exhibited enhanced survival, indicating a protective role of *Achl* knockdown under oxidative stress. *n* = 300 per group. Data are from three biological repeats. I) qPCR validation of *Achl* knockdown efficiency in *Elav‐GAL4*; *UAS‐dicer2/Achl^RNAi^* flies, ^***^ *p* < 0.001 by two‐tailed unpaired t‐test. Data are from four biological repeats. J) Circadian expression profile of *Achl* mRNA in *iso^31^* and *clk^out^* flies. *Achl* exhibited rhythmic expression in controls (peak at ZT21), which was abolished in *clk^out^* mutants. Rhythmicity was assessed using JTK‐CYCLE; data were normalized to *iso^31^* ZT1 expression levels. mean ± SEM; two‐tailed unpaired t‐test for statistical comparisons, ^**^ *p* < 0.01, ^***^ *p* < 0.001, ^****^ *p* < 0.0001. K–M) Dual‐luciferase reporter assays demonstrating CLK‐dependent transcriptional activation of *Achl* via E‐box elements in its promoter. K) Schematic showing wide‐type and mutated E‐box sites in the *Achl* promoter. L) Luciferase assays revealed that CLK significantly activates *Achl* transcription. ^*^ *p* < 0.05, ^**^ *p* < 0.01, ^***^ *p* < 0.001 by one‐way ANOVA with Tukey's post hoc test. Data are from two biological repeats. M) Mutagenesis of E‐box sequences abolished CLK‐induced activation, confirming direct regulation via E‐box motifs. Statistical significance indicated by *p* values (^****^ *p* < 0.0001 by one‐way ANOVA with Tukey's post hoc test. Data are from three biological repeats.

CLK regularly functions as a transcriptional activator; given the repressive role of CLK in basal immune gene expression, we next sought to identify downstream effectors of CLK. Transcriptomic profiling revealed *Achilles* (*Achl*), a previously identified circadian output gene, as one of the top downregulated genes in *clk^out^* flies (Figure 2F). Consistent with this, reanalysis of the published transcriptome data^[^ ^37^ ^]^ from pan‐neuronal *Achl* knockdown flies (*Elav‐GAL4*;; *UAS‐dicer2*/*Achl^RNAi^*) showed robust upregulation of AMP genes (Figure S4A,B, Supporting Information), resembling the immune activation profile observed in *clk^out^* flies (Figure S4C, Supporting Information). Next, we performed comparative transcriptomic analysis between *clk^out^* and *Achl*‐deficient flies, and identified 65 overlapping DEGs (**Figure** **3**A; Figure S4D, Supporting Information), many of which are enriched in immune and stress‐response pathways. Pathway enrichment analyses revealed shared activation of the Toll, IMD, and humoral immune signaling pathways (Figure 3B,C). Chord diagram visualization demonstrated convergence on functional categories such as Gram‐positive bacterial defense and humoral antimicrobial responses, with central nodes including *AttA*, *Mtk*, and *Drs* (Figure 3D). These results support a shared transcriptional program linking CLK and ACHL in the regulation of innate immune responses. Temporal profiling of AMP gene expression revealed that *clk^out^* flies exhibit both elevated and dysregulated AMP induction compared to controls (Figure 3E,F; Figure S4E, Supporting Information), reinforcing the notion that CLK suppresses innate immune activation under basal conditions. Notably, pan‐neuronal knockdown of *Achl* also resulted in robust upregulation of multiple AMPs—including *Mtk*, *Drs*, *Dro*, and *AttA*—mimicking the transcriptional phenotype of *clk^out^* flies (Figure 3G). These parallel outcomes suggest that *Achl* may act downstream of CLK in restraining immune gene expression. Supporting this, *Achl* transcript levels were markedly reduced in *clk^out^* flies (Figure S4F, Supporting Information), while *clk* expression remained unchanged upon *Achl* knockdown (Figure S4G, Supporting Information), indicating a unidirectional regulatory relationship whereby CLK positively regulates *Achl* to maintain immune homeostasis.

Next, we examined whether *Achl* plays a similar role in oxidative stress resistance. We used *Elav‐GAL4* to knock down *Achl* pan‐neuronally and checked the survival upon PQ exposure. Indeed, loss of *Achl* significantly improved survival under oxidative stress (Figure 3H,I), phenocopying *clk^out^* flies. To further investigate the regulation of *Achl* by the circadian clock, we examined its temporal expression pattern under LD conditions. Consistent with the previous report, *Achl* mRNA exhibited robust circadian oscillation, peaking at ZT21 in wild‐type flies (Figure 3J). In contrast, both the rhythmic expression pattern and amplitude of *Achl* were markedly diminished in *clk^out^* mutants (Figure 3J), indicating that CLK regulates the circadian transcription of *Achl*. CLK activates downstream clock‐controlled gene expression via binding of E‐box motifs (CACGTG). So, we examined the *Achl* promoter for potential CLK‐binding sites. Promoter Sequence analysis of the *Achl* promoter revealed the presence of multiple canonical E‐box elements (Figure 3K; Figure S4H, Supporting Information), suggesting a potential direct regulation. To functionally validate this interaction, we performed dual‐luciferase reporter assays. Overexpression of CLK led to dose‐dependent activation of the *Achl* promoter in vitro (Figure 3L; Figure S4I, Supporting Information). Moreover, mutation of E‐box motifs significantly reduced luciferase activity, demonstrating that CLK activates *Achl* transcription via these regulatory elements (Figure 3M). Collectively, these findings establish *Achl* as a pivotal rhythmic factor acting downstream of CLK to orchestrate innate immune restraint and promote organismal resilience under oxidative stress.

### 2.4 ACHL Post‐Transcriptionally Suppresses *Relish* and Downstream AMP Production

**Figure 4:**
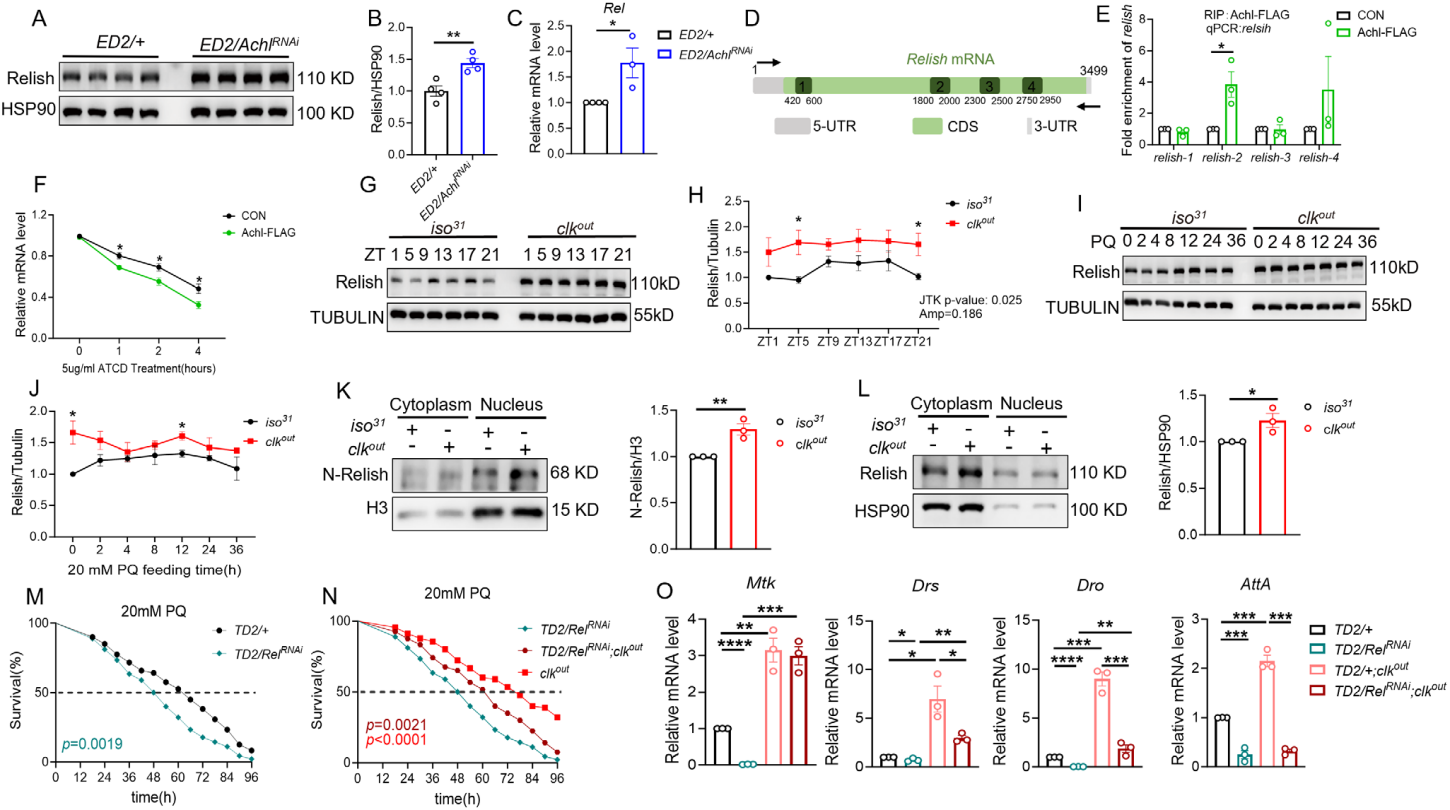
ACHL modulates immune responses to oxidative stress by regulating *Relish* mRNA stability. A,B) Western blot and densitometric quantification showing increased Relish protein levels at ZT1 in *Elav‐GAL4*; *UAS‐dicer2/Achl^RNAi^* flies compared to controls, indicating that ACHL negatively regulates Relish abundance. Data represent four independent replicates; mean ± SEM; ^**^ *p* < 0.01 by two‐tailed unpaired t‐test. C) qRT‐PCR analysis showing significantly elevated *Relish* mRNA levels in *ED2/Achl^RNAi^* flies compared to controls (^*^ *p* < 0.05 by two‐tailed unpaired t‐test.), indicating negative regulation of *Relish* by ACHL. D) Schematic representation of Relish mRNA transcript structure and qPCR primer positions. E) RNA immunoprecipitation followed by qPCR (RIP–qPCR) confirms that ACHL binds specific regions of *Relish* mRNA, with the strongest enrichment detected in Region 2. ^*^ *p* < 0.05 by two‐tailed unpaired t‐test. Data are from three biological repeats. F) Overexpression of ACHL in S2 cells reduces *Relish* mRNA stability. Data are mean ± SEM from three biological replicates; ^*^ *p* < 0.05 by two‐tailed unpaired t‐test. G,H) Western blot analysis of Relish protein across ZT in *iso^31^* and *clk^out^* flies. While Relish shows circadian rhythmicity in controls, this pattern is disrupted in *clk* mutants. Rhythmicity was assessed using JTK‐CYCLE. Statistical significance was assessed using a two‐tailed unpaired t‐test (^*^ *p* < 0.05). Data are from three biological repeats. I–J) PQ‐induced Relish protein abundance is higher and more sustained in *clk^out^* flies than in *iso^31^* controls. Quantification is normalized to *iso^31^* at ZT0. Statistical significance was assessed using a two‐tailed unpaired t‐test (^*^ *p* < 0.05). Data are from three biological repeats. K,L) Subcellular distribution of Relish protein. Depletion of *clk* increases nuclear N‐terminal Relish (N‐Relish) levels K) and alters full‐length Relish distribution in the cytoplasm L). mean ± SEM from three biological replicates. ^*^ *p* < 0.05, ^**^ *p* < 0.01 by two‐tailed unpaired *t*‐test. M) Knockdown of *Relish* in circadian cells (*tim‐GAL4*, *UAS‐dicer2/Relish^RNAi^*) reduces survival under PQ stress, indicating its functional importance in oxidative stress adaptation (*n* = 90–120). Data are from three biological repeats. N) Combined knockdown of *clk* and *Relish* in clock neurons reduces survival compared to *clk^out^* alone but remains higher than *Relish‐*RNAi alone, indicating partial epistasis (*n* = 90–120; log‐rank test). Data are from three biological repeats. O) qPCR analysis of AMP genes (*Mtk*, *Drs*, *Dro*, *AttA*) in *Relish‐*RNAi flies with or without *clk* mutation. Data are mean ± SEM; *p*‐values as indicated, ^*^ *p* < 0.05, ^**^ *p* < 0.01, ^***^ *p* < 0.001, ^****^ *p* < 0.0001 by two‐tailed unpaired *t*‐test. Data from three biological repeats.

The observation of immune hyperactivation in both *Achl*‐deficient and *clk^out^* flies prompted us to explore the downstream effectors linking the CLK–ACHL pathway to immune responses. Relish, the *Drosophila* NF‐κB homolog and terminal effector of the IMD pathway, emerged as a likely candidate. In flies with *Achl* knockdown, both Relish mRNA and protein levels were significantly elevated (**Figure** **4**A–C), indicating that ACHL acts as a negative regulator of Relish abundance.

Given that *Achl* encodes an RNA‐binding protein and its deficiency leads to elevated Relish expression, we hypothesized that ACHL may post‐transcriptionally regulate Relish by directly binding its mRNA. To explore this possibility, we first conducted predictive RNA–protein interaction analyses by RNA‐Protein Interaction Prediction (RPISeq),^[^ ^39^, ^40^ ^]^ which revealed a putative interaction between ACHL and *Relish* mRNA (Figure S5A, Supporting Information). Consistently, AlphaFold3‐based structural modeling identified multiple potential ACHL‐binding sites along the *Relish* transcript (Figure S5B, Supporting Information), suggesting a direct post‐transcriptional regulatory interface. These predictions support a model in which ACHL binds to *Relish* mRNA to modulate its stability. Next, we performed RNA immunoprecipitation followed by qPCR (RIP–qPCR) by overexpression of a tagged version of ACHL and validated direct binding of ACHL to *Relish* mRNA at multiple predicted sites, with region 2 (1800–2000 bp) showing the highest enrichment (Figure 4D,E). Furthermore, RNA stability analysis demonstrated that overexpression of ACHL led to reduced *Relish* mRNA levels and decreased transcript stability (Figure 4F; Figure S5C, Supporting Information), consistent with the prediction that ACHL acts as an RNA destabilizer. Indeed, qPCR profiling of *Relish* expression in the fly head confirmed increased transcript abundance in the *clk^out^* (Figure S5D, Supporting Information). Lastly, western blot analysis further revealed elevated Relish protein levels under both basal and PQ‐treated conditions in *clk^out^* flies (Figure 4G–J; Figure S5E,F, Supporting Information). These findings establish *Relish* as a key downstream effector of the CLK–ACHL axis, whereby loss of *Achl*‐mediated post‐transcriptional repression leads to sustained Relish activation and immune gene overexpression.

Relish, which functions as a transcription factor, needs to translocate into the nucleus to exert transcriptional control over AMP genes. We next assessed N‐terminal Relish (N‐Relish, active) protein levels. Nuclear N‐Relish accumulation was significantly higher in *clk^out^* flies than in controls (Figure 4K), indicating enhanced NF‐κB signaling. Interestingly, full‐length cytoplasmic Relish was also elevated (Figure 4L), suggesting dysregulation of both activation and processing dynamics. Functionally, circadian neuronal knockdown of *Relish* using *tim‐GAL4*, *UAS‐dicer2* sensitized flies to PQ, leading to reduced survival compared to controls (Figure 4M). Importantly, simultaneous depletion of *clk* and *Relish* produced an intermediate survival phenotype—significantly lower than *clk^out^* but improved relative to *Relish* knockdown alone (Figure 4N). This genetic interaction supports *Relish* as a downstream effector mediating the protective phenotype observed in *clk^out^* flies. In parallel, AMP gene expression was substantially reduced in *Relish*‐depleted flies (Figure 4O), further confirming its essential role in AMP gene induction. Consistent with the proposed regulatory hierarchy, RNAi‐mediated knockdown of *Relish* significantly reduced its mRNA levels in both *iso^31^* and *clk^out^* backgrounds (Figure S5G,H, Supporting Information). In contrast, *clk* expression remained unaffected, and *Achl* expression exhibited only a modest increase upon *Relish* depletion (Figure S5I, Supporting Information), further supporting a unidirectional regulatory model in which CLK and ACHL function upstream of *Relish*.

Together, these findings define a circadian‐regulated post‐transcriptional mechanism in which CLK drives *Achl* expression, and ACHL suppresses *Relish* via direct mRNA destabilization. Disruption of this regulatory axis leads to Relish accumulation, overactivation of AMP genes, and enhanced stress resistance, positioning ACHL as a critical RNA‐binding repressor at the intersection of circadian and immune pathways.

### 2.5 The CLK–ACHL*–Rel* Axis Establishes a Circadian Checkpoint for Immune Regulation Under Oxidative Stress

**Figure 5:**
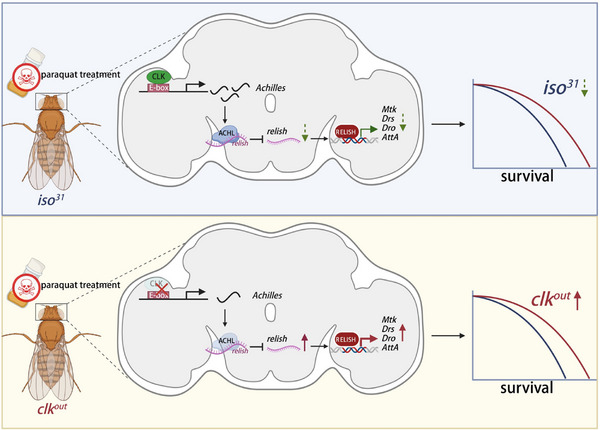
CLK regulates immune homeostasis and oxidative stress resilience via the ACHL–*Relish* axis in *Drosophila*. Schematic representation of the proposed mechanism by which CLK modulates immune responses and survival upon PQ‐induced oxidative stress. In *iso^31^* (top), CLK activates *Achl* transcription by binding to the E‐box elements in its promoter. ACHL, in turn, binds to *Relish* mRNA and promotes its degradation, thereby reducing Relish protein accumulation and suppressing downstream AMP gene expression. This pathway maintains immune quiescence and promotes organismal survival under oxidative stress. In *clk^out^* flies (bottom), loss of *clk* results in reduced *Achl* expression, increased *Relish* mRNA stability, and heightened AMP production. The resulting immune activation contributes to enhanced resistance to oxidative stress, albeit at the cost of circadian regulation. This model illustrates the role of the CLK–ACHL–*Relish* axis as a circadian checkpoint that fine‐tunes immune output and redox homeostasis in response to environmental stress.

To consolidate the mechanistic insights uncovered in this study, we propose a model in which the circadian transcription factor CLK orchestrates immune regulation and oxidative stress adaptation through a post‐transcriptional axis involving ACHL and the NF‐κB homolog *Relish* (**Figure** **5**). In wild‐type flies, CLK binds to E‐box motifs in the *Achl* promoter, thereby driving its rhythmic transcription. The resulting ACHL protein interacts with *Relish* mRNA to promote its degradation. This post‐transcriptional repression limits Relish protein accumulation and consequently suppresses the transcription of downstream AMP genes such as *Mtk*, *Drs*, *Dro*, and *AttA*. This multilayered circadian regulation serves to constrain immune activation under oxidative challenge, thus preserving immune homeostasis.

Disruption of *clk* abrogates this regulatory axis by downregulating *Achl*, resulting in the stabilization of *Relish* transcripts, accumulation of both full‐length and cleaved nuclear Relish, and subsequent overactivation of AMP gene expression. Paradoxically, this immune hyperactivation is associated with enhanced survival in *clk^out^* flies under oxidative stress conditions. These results suggest that in the absence of circadian gating, derepression of immune effectors may act as a compensatory mechanism that augments host tolerance to oxidative damage.

Collectively, our findings define a circadian checkpoint wherein CLK modulates immune tone and redox adaptation via ACHL‐mediated post‐transcriptional repression of *Relish*. This CLK–ACHL–*Rel* regulatory cascade exemplifies a temporal interface between the circadian clock and innate immune signaling, revealing that disruption of clock components can reprogram stress responsiveness by reshaping immune homeostasis.

### 2.6 CLOCK Promotes Oxidative Stress Sensitivity in Mammalian Cells

To evaluate whether the regulatory function of CLK in oxidative stress adaptation is evolutionarily conserved, we turned to a mammalian cell model. NIH3T3 cells were exposed to increasing concentrations of H_2_O_2_, which elicited a dose‐dependent accumulation of intracellular ROS as detected by DCFH‐DA fluorescence staining (Figure S6A, Supporting Information). This ROS elevation was accompanied by a marked reduction in cell viability, assessed by MTT assay (Figure S6A, Supporting Information), confirming successful induction of oxidative stress. Consistently, flow cytometric analysis using a FITC‐based ROS probe further confirmed a graded increase in fluorescence intensity with rising H_2_O_2_ doses (Figure S6C, Supporting Information), reinforcing the reliability of the oxidative challenge model (Figure S6C, Supporting Information). Using this model, we next examined whether CLOCK contributes to oxidative stress sensitivity. Comparative analysis between wild‐type and *Clock*‐knockout (*Clock* ^−/−^) NIH3T3 cells revealed that *Clock^−/−^* cells retained significantly higher viability following exposure to 600 µm H_2_O_2_ (Figure S6D, Supporting Information). These results suggest that loss of *Clock* confers a cytoprotective advantage under oxidative insult. The enhanced oxidative resilience observed in *Clock^−/−^* cells mirrors the phenotype of *clk*‐deficient *Drosophila*, where loss of circadian transcriptional input improves survival under oxidative stress. We also investigated the regulation of *Larp6* and *Larp7* (mammalian orthologs of *Drosophila Achl*) in mammalian cells. Quantitative PCR revealed that the mRNA levels of both *Larp6* and *Larp7* were significantly reduced in *Clock^−/−^* versus WT control cells (Figure S6E, Supporting Information), suggesting CLOCK regulates these genes in mammalian cells. Furthermore, to examine whether CLOCK regulates *LARP6* and *LARP7* transcription, we assessed BMAL1/CLOCK transactivation on their promoters using dual‐luciferase reporter assays in HEK293T cells. Co‐expressing BMAL1/CLOCK strongly increased luciferase activity for both promoters (Figure S6F, Supporting Information), indicating BMAL1/CLOCK regulates *LARP6* and *LARP7* in a promoter‐dependent manner.

Collectively, these findings support an evolutionarily conserved role for CLOCK/CLK in sensitizing cells to redox stress and highlight circadian transcription factors as critical modulators of oxidative homeostasis across species.

## 3 Discussion

Chronic oxidative stress leads to excessive accumulation of ROS that damages biomolecules such as lipids, proteins, and DNA.^[^ ^41^ ^]^ Oxidative stress occurs when the equilibrium between the generation of ROS and the cellular antioxidant defense system is disturbed. This redox imbalance contributes to the development of multiple chronic diseases, including cardiovascular disorders, neurodegenerative diseases, and cancer.^[^ ^42^ ^]^ Circadian clock genes are increasingly recognized as pivotal regulators of redox homeostasis;^[^ ^43^, ^44^ ^]^ however, the molecular mechanisms underlying this regulatory interplay remain elusive.

In this study, we define a previously uncharacterized circadian–immune–redox axis orchestrated by the circadian transcription factor CLK and the RNA‐binding protein Achilles (ACHL) in *Drosophila*. Loss of *clk* enhances resistance to PQ‐induced oxidative stress, accompanied by elevated basal AMP expression and sustained Rel/NF‐κB signaling. Mechanistically, CLK drives the rhythmic expression of *Achl* through the canonical E‐box motifs. ACHL post‐transcriptionally regulates *Relish* by repressing its mRNA stability. Depletion of *clk* or *Achl* leads to increased Rel abundance, enhanced AMP gene transcription, and heightened oxidative‐stress resistance. These findings uncover a novel post‐transcriptional checkpoint integrating circadian cues with innate immunity and oxidative stress adaptation. Our findings also broaden the conceptual framework of circadian regulation by highlighting ACHL as a temporal effector acting at the RNA level of target genes. While many studies have focused on transcriptional feedback loops, this study emphasizes RNA stability as a regulatory layer downstream of circadian transcription factors, adding depth to our understanding of how clocks shape stress physiology.

Despite these mechanistic insights, several questions remain. First, although *clk* or *Achl* deficiency enhances AMP gene expression in the fly head under PQ‐induced oxidative stress, not all upregulated AMPs are direct Relish targets—for example, *Drosomycin* is Toll‐dependent and largely Relish‐independent—indicating broad NF‐κB activation rather than selective IMD engagement. Here, our data demonstrate that ACHL reduces *Relish* transcript stability, but the precise biochemical underpinnings of this repression remain to be elucidated. It is unclear whether ACHL exerts its effect by recruiting specific RNA decay machineries such as deadenylases or exonucleases, or by binding distinct sequence motifs within the *Relish* transcript. Detailed mechanistic dissection using RNA immunoprecipitation sequencing (RIP‐seq), 3′UTR truncation analysis, and decay kinetics assays will help uncover the RNA elements and protein partners involved.

Second, how do neuronal changes in CLK‐ACHL affect fly survival under oxidative stress? Our gene expression analyses were restricted to the head—where circadian and immune pathways intersect. Although AMP induction reflects immune activation, AMPs do not directly neutralize ROS. Thus, the enhanced oxidative stress resistance observed in *clk* or *Achl* mutants likely arises from immune‐mediated systemic homeostasis rather than improved antioxidant defense. Future studies employing tissue‐specific perturbations and inter‐organ reporter systems will be instrumental in delineating how neuronal circadian cues coordinate organism‐wide immune responses.

Third, our results indicate that AMP expression serves as a proxy for Relish activity, which is modulated by the CLK–ACHL–Relish axis in the context of oxidative stress and lifespan regulation. Under baseline conditions, *clk* mutant flies exhibit increased AMP expression and reduced longevity, suggesting that chronic immune activation imposes physiological costs potentially linked to tissue stress, proteostasis disruption, and the activation of pro‐aging signaling cascades. In contrast, under paraquat‐induced oxidative stress, AMP levels remain elevated, but lifespan is paradoxically extended in *clk* mutants, indicating a beneficial role of AMP overexpression in mitigating acute oxidative insults. This apparent discrepancy implies a dynamic trade‐off whereby the adaptive advantage of immune activation in acute stress conditions may turn deleterious when sustained chronically. Future studies that combine genetic AMP suppression with lifespan and stress assays in *clk*‐deficient backgrounds will be instrumental in resolving these dualistic roles.

Additionally, our mammalian experiments have several limitations. First, NIH3T3 fibroblasts lack robust endogenous circadian oscillations unless synchronized by external cues, and thus represent a reductionist system to probe the contribution of CLOCK to oxidative stress sensitivity at the cellular level rather than a model of tissue‐ or organism‐level circadian regulation. Second, in mammals, the paralog NPAS2 can functionally compensate for CLOCK. For instance, circadian rhythms persist in *Clock^−/−^* fibroblasts but are abolished when NPAS2 is additionally depleted.^[^ ^45^ ^]^ Such genetic compensation may attenuate the phenotypic consequences of CLOCK deficiency in our NIH3T3 model and complicate the interpretation of oxidative stress responses. Finally, the conservation of this regulatory module across species and stress paradigms remains to be fully addressed. The observation that *Clock*‐deficient NIH3T3 cells exhibit improved survival under oxidative stress suggests that elements of the CLK–ACHL–*Relish* axis may be conserved. However, the roles of putative mammalian orthologs such as LARP6 and LARP7^[^ ^46^, ^47^ ^]^ in circadian or immune regulation remain underexplored. Cross‐species functional studies, coupled with investigations under diverse stress conditions—including bacterial infection, heat shock, and metabolic stress—are needed to determine the broader relevance and functional scope of this regulatory axis.

Collectively, this work establishes ACHL as a clock‐controlled RNA‐binding protein that modulates innate immunity and oxidative resilience via repression of *Relish* mRNA. By integrating temporal transcriptional signals with RNA‐level immune checkpoints, the CLK–ACHL–*Relish* axis fine‐tunes oxidative stress responses and cellular adaptation. These findings offer a mechanistic bridge between circadian biology, post‐transcriptional regulation, and redox immunology, providing a foundation for future studies on temporal regulation of immune homeostasis and its implications in aging and disease.

## 4 Experimental Section

### Fly Stocks and Locomotor Activity Analysis

All fly strains were maintained on standard cornmeal‐agar medium at 25 °C under a 12:12 h light‐dark (LD) cycle. The following GAL4 driver lines were used for tissue‐specific gene manipulation: *TG4* (*tim‐GAL4*), *PG4* (*PDF‐GAL4*), *TD2* (*tim‐GAL4*; *UAS‐dicer2*), *PD2* (*6*), *TD2; PDF‐GAL80*, *TD2; tubP‐GAL80^ts^*, *ED2* (*Elav‐GAL4*;; *UAS‐dicer2*). UAS‐RNAi and effector lines were obtained from the Vienna Drosophila Resource Center (VDRC), Bloomington Drosophila Stock Center, and Tsinghua Fly Center.

For behavioral assays, male flies (3–5 days old) were individually loaded into glass activity tubes and monitored using the *Drosophila* Activity Monitoring (DAM) system (TriKinetics, USA). Flies were entrained under LD for 4 days, followed by 6 days of constant darkness (DD). Light intensity during LD cycles was maintained at ≈500 lux. Raw activity data were processed using FaasX software,^[^ ^48^ ^]^ and actograms were generated and analyzed using the MATLAB.^[^ ^49^ ^]^

### Oxidative Stress Survival Assay

Adult flies (3–5 days old) were pre‐acclimated on standard food for 3 days and then transferred to food containing 20 mm paraquat (PQ, Aladdin; M106761‐1 g). Flies were maintained at 25 °C under a 12:12 h LD cycle, and survival was scored every 12 h. Flies unresponsive to gentle tapping were recorded as dead. Each condition included ≥60 flies across at least three independent replicates. In the survival experiments, the number of flies, along with their sex and genotype, is detailed in Table S1 (Supporting Information).

### RNA Extraction from Fly Heads

To isolate RNA from fly heads, whole flies were flash‐frozen in liquid nitrogen and vortexed to separate heads. Head tissues were manually sorted on a prechilled aluminum plate and transferred to 1.5 mL microcentrifuge tubes containing 100 µL TRIzol reagent (Thermo Fisher Scientific). Total RNA was extracted following the manufacturer's protocol. Homogenization was performed on ice using a handheld homogenizer (Beijing Lanjieke, L‐C119‐0974). Phase separation was achieved with 200 µL chloroform, followed by centrifugation at 12 000 rpm for 15 min at 4 °C. The aqueous phase was precipitated with an equal volume of isopropanol and incubated at −20 °C, followed by centrifugation. The RNA pellet was washed twice with 75% ethanol, air‐dried, and resuspended in DEPC‐treated water. RNA purity and concentration were measured using a NanoDrop spectrophotometer (Thermo Fisher Scientific).

### RNA Sequencing and Data Analysis

Fly heads from *iso^31^* and *clk^out^* flies were collected at 0 and 24 h postparaquat treatment for RNA sequencing. Total RNA was extracted and subjected to library preparation and Illumina sequencing (BMKCloud, Qingdao, China). Sequencing reads were mapped to the *Drosophila melanogaster* reference genome (Release 6 + IS01 MT). Differential gene expression was analyzed using DESeq2, with significance defined as *p* < 0.05 and |log_2_FC| > 1. Functional enrichment analyses (KEGG and GO) were performed via SangerBox^[^ ^50^ ^]^ and SRplot.^[^ ^51^ ^]^ Three biological replicates were included per condition.

### Plasmid Construction

The CDS region of the target gene was amplified from adult fly head cDNA using high‐fidelity polymerase and gene‐specific primers. PCR products were gel‐purified and cloned into linearized pAc‐FLAG or pGL3 vectors via homologous recombination (Exnase MultiS, Vazyme). Vector linearization was achieved by double digestion with restriction enzymes, followed by gel purification. Recombinant plasmids were transformed into competent *E. coli* and screened by colony PCR. Positive clones were verified by Sanger sequencing. Verified constructs were cultured, and plasmids were extracted using commercial kits according to the manufacturer's instructions.

### Cell Culture and Transfection

*Drosophila* S2 cells were cultured in Schneider's Insect Medium (Sigma‐Aldrich) supplemented with 10% fetal bovine serum (FBS) at 25 °C. Cells were transfected using Cellfectin reagent (Thermo Fisher Scientific) according to the manufacturer's instructions. The plasmids used included pAc‐EGFP, pAc‐*Achl*‐FLAG, pAc‐*clk*‐V5, pGL3‐per‐promoter, pGL3‐*Achl*‐promoter, and pGL3‐*Achl*‐promoter‐mut.

For the mRNA stability assay, cells were transfected with 2.0 µg of pAc‐*Achl*‐FLAG, incubated for 48 h, and then treated with 10 µg mL^−1^ actinomycin D (ATCD; Sigma‐Aldrich) for 0, 1, 2, or 4 h prior to RNA extraction and qPCR analysis.

For luciferase reporter assays, S2 cells were co‐transfected with 50 ng of pGL3‐per‐promoter or pGL3‐*Achl*‐promoter constructs, pAc‐Renilla (internal control), and varying amounts of pAc‐*clk*‐V5 or pGL3‐*Achl*‐promoter‐mut plasmids. Luciferase activity was measured at 48 h post‐transfection using the Dual‐Luciferase Reporter Assay System (Promega), and firefly luciferase signals were normalized to Renilla.

### ROS Detection via DCFH‐DA Staining

Following LD entrainment for three days, adult fly brains were dissected in 1× PBS on ice. Brains were incubated with 10 µm DCFH‐DA (Beyotime, S0033S) at room temperature for 1 h, followed by three PBS washes. Fluorescence imaging was performed using a laser‐scanning confocal microscope (Leica SP8 LIGHTING).

### Western Blot Analysis

Protein lysates were prepared from dissected fly heads (20–30 per sample) or cultured S2 cells using RIPA buffer supplemented with protease and phosphatase inhibitors. Brain tissues were homogenized on ice, incubated for 30 min at 4 °C, and centrifuged at 12 000 rpm for 15 min. S2 cells were pelleted at 5000 rpm for 3 min prior to lysis. Protein samples were denatured at 99 °C for 10 min, resolved by SDS‐PAGE, and transferred to PVDF membranes (Millipore) at 200 mA for 2 h. Membranes were blocked in 5% nonfat milk in TBST for 1 h, incubated overnight at 4 °C with primary antibodies, followed by 1 h incubation with HRP‐conjugated secondary antibodies. Signals were visualized using enhanced chemiluminescence (ECL). TUBULIN (Proteintech;11224‐1‐AP); HSP90 (Proteintech;13171‐1‐AP); Relish (DSHB; AB‐1553772); N‐Relish (Raybiotech; RB‐14‐00040).

### Reverse Transcription and Quantitative PCR (qPCR)

One microgram of total RNA was treated with gDNA wiper mix (Vazyme, R123‐01) at 42 °C for 2 min and reverse‐transcribed using HiScript Q RT SuperMix (Vazyme). qPCR was performed using ChamQ Universal SYBR qPCR Master Mix (Vazyme, Q711‐02) under the following cycling conditions: 95 °C for 3 min; 40 cycles of 95 °C for 10 s and 60 °C for 30 s. Relative mRNA expression was calculated using the 2^‐ΔΔCt method, with *rpl32* as the internal reference. The primers used in the experiments are listed in Table S2 (Supporting Information).

### Nuclear and Cytoplasmic Protein Fractionation

To isolate nuclear and cytoplasmic proteins, ≈20 mg of fly heads were homogenized in Cytoplasmic Extraction Buffer A (Beyotime, E101‐01) containing protease and phosphatase inhibitors. After adding Buffer B, lysates were vortexed and centrifuged to collect the cytoplasmic fraction. The pellet was further washed with Buffer A and resuspended in Nuclear Extraction Buffer. After repeated vortexing and incubation on ice, samples were centrifuged to isolate nuclear proteins. Extracted proteins were mixed with SDS loading buffer, denatured at 99 °C, and stored at −80 °C until use.

### RNA Immunoprecipitation (RIP)

S2 cells were transfected for 48 h and then lysed in RIP lysis buffer (3 mL per sample). Following centrifugation at 2500 × g for 15 min at 4 °C, the supernatant was incubated overnight with either anti‐FLAG (Sigma; F18/04) antibody or IgG (Proteintech; 10283‐1‐AP) control, followed by incubation with protein A/G magnetic beads. Beads were extensively washed with RIP buffer and PBS, then resuspended in proteinase K digestion buffer for 30 min at 55 °C. RNA was extracted using TRIzol, reverse‐transcribed, and subjected to qPCR using relish‐specific primers. The primers used in the experiments are listed in Table S2 (Supporting Information).

### Catalase (CAT) Activity Assay

Catalase (CAT) activity was measured in fly heads using a commercial hydrogen peroxide assay kit (Beyotime Biotechnology, Cat. No. S0051) following the manufacturer's instructions. Adult flies were collected at 0, 12, and 24 h after paraquat (PQ) feeding. Heads were separated on dry ice and homogenized in the kit lysis buffer. H_2_O_2_ standards were prepared from the supplied 1 m stock solution, and a standard curve was generated. For each sample, 50 µL of homogenate was loaded in triplicate wells and incubated at 25 °C for 30 min, after which absorbance was measured at 560 nm. Total protein concentration was determined by the BCA assay (Beyotime), and CAT activity was calculated according to the manufacturer's formula and expressed as units per mg protein.

### Structural Modeling of Protein–mRNA Interactions Using AlphaFold 3

To explore the structural basis of RNA‐binding protein (RBP)–mRNA interactions, we employed AlphaFold 3 for predictive modeling of the protein–RNA complex. The amino acid sequence of the target protein was retrieved from the UniProt database (https://www.uniprot.org/) and submitted to the AlphaFold Protein Structure Database (https://alphafold.ebi.ac.uk/) for structure prediction.^[^ ^52^ ^]^ For mRNA modeling, the full‐length nucleotide sequence or specific binding region of the target transcript (e.g., CDS or 3′UTR fragment) was input along with the protein sequence into the AlphaFold 3 multimer framework.

The resulting predicted protein–RNA complex structure was exported in PDB format and visualized using PyMOL (The PyMOL Molecular Graphics System, Version 2.6, Schrödinger, LLC). Key contact residues and RNA‐binding interfaces were identified based on spatial proximity and surface accessibility. Interaction sites were highlighted using cartoon and stick representations. High‐resolution structural images were generated using ray tracing and exported as PNG files for subsequent figure preparation.

### Statistical Analysis

Rhythmicity analysis was performed using the JTK_CYCLE^[^ ^53^ ^]^ algorithm in R, with the circadian period set to 24 h. Phase and amplitude estimates were derived as previously described. Survival analysis was conducted using the log‐rank (Mantel–Cox) test. Gene expression was analyzed using the ΔΔCt method, normalized to *rpl32*. Additional normalization strategies—using either control group values or the median across time points—were applied where appropriate, as detailed in figure legends. Western blot quantification was conducted using ImageJ (FIJI), and data were normalized to loading controls for statistical comparison. All data are presented as mean ± SEM. Statistical analyses were performed using GraphPad Prism 10.0 software. For comparisons between two groups, a two‐tailed unpaired *t*‐test was used. For multiple group comparisons, one‐way ANOVA was applied, followed by Tukey's post‐hoc test. A *p*‐value of less than 0.05 was considered statistically significant.

## Conflict of Interest

The authors declare no conflict of interest.

## Author Contributions

X.L. and J.F. contributed equally to this work. X.L., L.P., and Y.Z. conceived the study and designed the experiments. X.L., J.F., D.C., W.L., and Y.L. performed the experiments. X.L., J.F., and Y.Z. analyzed the data and wrote the manuscript. All authors reviewed and approved the final version of the manuscript.

## Supporting information



Supporting Information

Supplemental Table 1

Supplemental Table 2

## Data Availability

All study data are included in the article and supplementary materials. All raw data, details of methods, analytical tools, and scripts are available upon reasonable request to the corresponding author.
