# Supplementary material for: The Circadian Transcription Factor CLOCK Modulates Oxidative Stress Resistance via the ACHL–Relish Axis in Drosophila
Source: Adv Sci (Weinh). 2025 Oct 30;13(4):e14388. doi: 10.1002/advs.202514388 (PMC12822383; doi:10.1002/advs.202514388)
Supplement: Supplementary file 1 — Supporting Information [file ADVS-13-e14388-s002.docx]

**Supplementary Materials for**

**The Circadian Transcription Factor CLOCK Modulates Oxidative Stress Resistance via the ACHL–*Relish* axis in Drosophila**

Xu Liu^1^*, Jiajia Fang ^1^*, Dawei Cheng^1^, Wei Luan ^1^, Yan Lv^2^, Wen Hu^1^, Lei Pan^2^, Yong Zhang^1✉^

^1^Jiangsu Key Laboratory of Drug Discovery and Translational Research for Brain Diseases, Cambridge-Suda Genomic Resource Center, The Fourth Affiliated Hospital, Suzhou Medical College, Soochow University, Suzhou 215123, Jiangsu, China.

^2^State Key Laboratory of Immune Response and Immunotherapy, Shanghai lnstitute of lmmunity and Infection, Chinese Academy of Sciences, Shanghai 200031, China

*Contributed equally to this work

^✉^Correspondence to yong.zhang@suda.edu.cn

Figure S1. Loss of *clk* enhances oxidative stress survival in *Drosophila*.

Figure S2. CLK regulates AMP gene expression under oxidative and infectious stress.

Figure S3. *Clk* deficiency alters redox homeostasis and enhances antioxidant responses.

Figure S4. CLK and ACHL coordinately regulate circadian expression of AMP genes in *Drosophila*.

Figure S5. Relish functions downstream of the CLK–ACHL axis to regulate oxidative stress responses in *Drosophila*.

Figure S6. Loss of *Clock* confers resistance to oxidative stress in NIH3T3 cells.


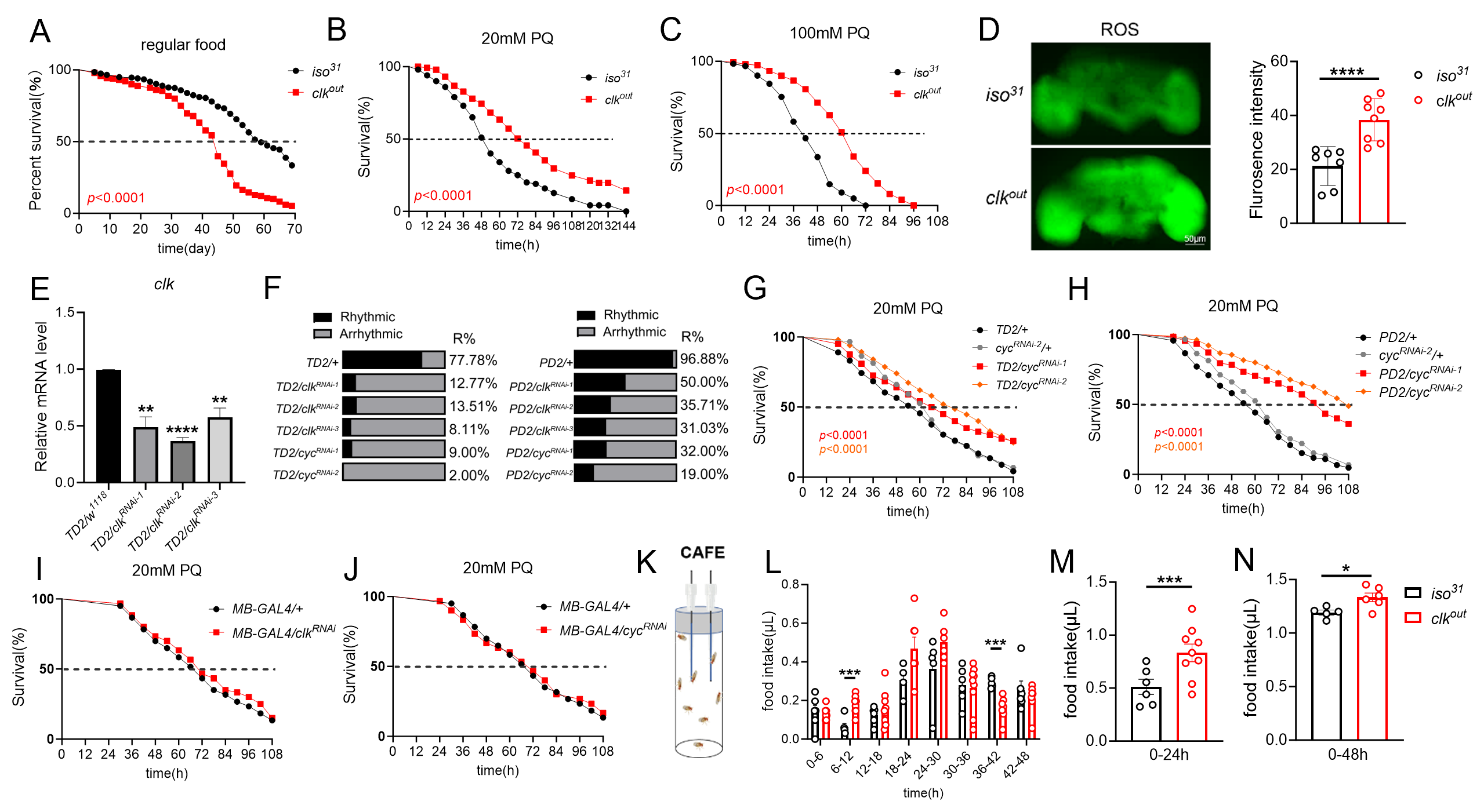


**Figure S1. Loss of *clk* enhances oxidative stress survival in *Drosophila*.**

(A) Lifespan curves of wild-type *iso^31^* and *clk^out^* flies maintained on regular food. *clk^out^* flies display a significantly shorter median lifespan compared to controls (log-rank test, *p* < 0.0001).
(B–C) Survival curves of *iso^31^* and *clk^out^* flies treated with paraquat (PQ) at 20 mM (B) or 100 mM (C). Under both oxidative stress conditions, *clk^out^* flies consistently survived longer than controls (log-rank test, *p* < 0.0001). (20 mM: *iso^31^* T50 = 54 h, *clk^out^* T50 = 78 h; 100 mM: *iso^31^* T50 = 42 h, *clk^out^* T50 = 66 h, T50 stands for median survival). Data are from two biological replicates.

(D) Left: Representative images of ROS accumulation in the brains of *iso^31^* and *clk^out^* flies, visualized using DCFH-DA staining. Right: Quantification of brain ROS levels based on fluorescence intensity (*iso^31^*, n = 7; *clk^out^*, n = 8). Scale bar: 50 μm. ****P < 0.0001. Data are from two biological replicates.

(E) qPCR analysis confirming effective knockdown of *clk* mRNA in *tim-GAL4*; *UAS-dicer2/clk^RNAi^* flies relative to controls. **P < 0.01; ****P < 0.0001 by one-way ANOVA with Tukey’s post hoc test. Data are from three biological replicates.

(F) Percentage of flies exhibiting circadian rhythmicity following *clk* or *cyc* knockdown in all circadian neurons (*TD2= tim-GAL4*; *UAS-dicer2*) or PDF-positive neurons (*PD2= PDF-GAL4*; *UAS-dicer2*) neurons, based on locomotor activity under constant darkness. Data are from two biological replicates.

(G–H) Survival curves of flies with *cyc* knockdown in all circadian neurons (*TD2= tim-GAL4*; *UAS-dicer2*, G) or in PDF-positive neurons (*PD2= PDF-GAL4*; *UAS-dicer2*, H) under 20 mM PQ treatment. Knockdown in both neuronal populations significantly improved survival. Data are from three biological replicates.

(I–J) Survival analysis of flies with *clk* or *cyc* knockdown in mushroom body neurons (*MB-GAL4*) under 20 mM PQ exposure. No significant survival benefit was observed, suggesting regional specificity in circadian regulation of oxidative stress responses. Data are from three biological replicates.

(K–N) Feeding behavior analysis using the capillary feeder (CAFE) assay. (K) Schematic diagram of the CAFE setup. (L–N) Quantification of total food intake over 24 hours (M) and 48 hours (N), showing significantly increased consumption in *clk^out^* flies compared to *iso^31^* controls. *P < 0.05, **P < 0.01, ****P < 0.0001 by two-tailed unpaired t-test. Data are from three biological replicates.

.


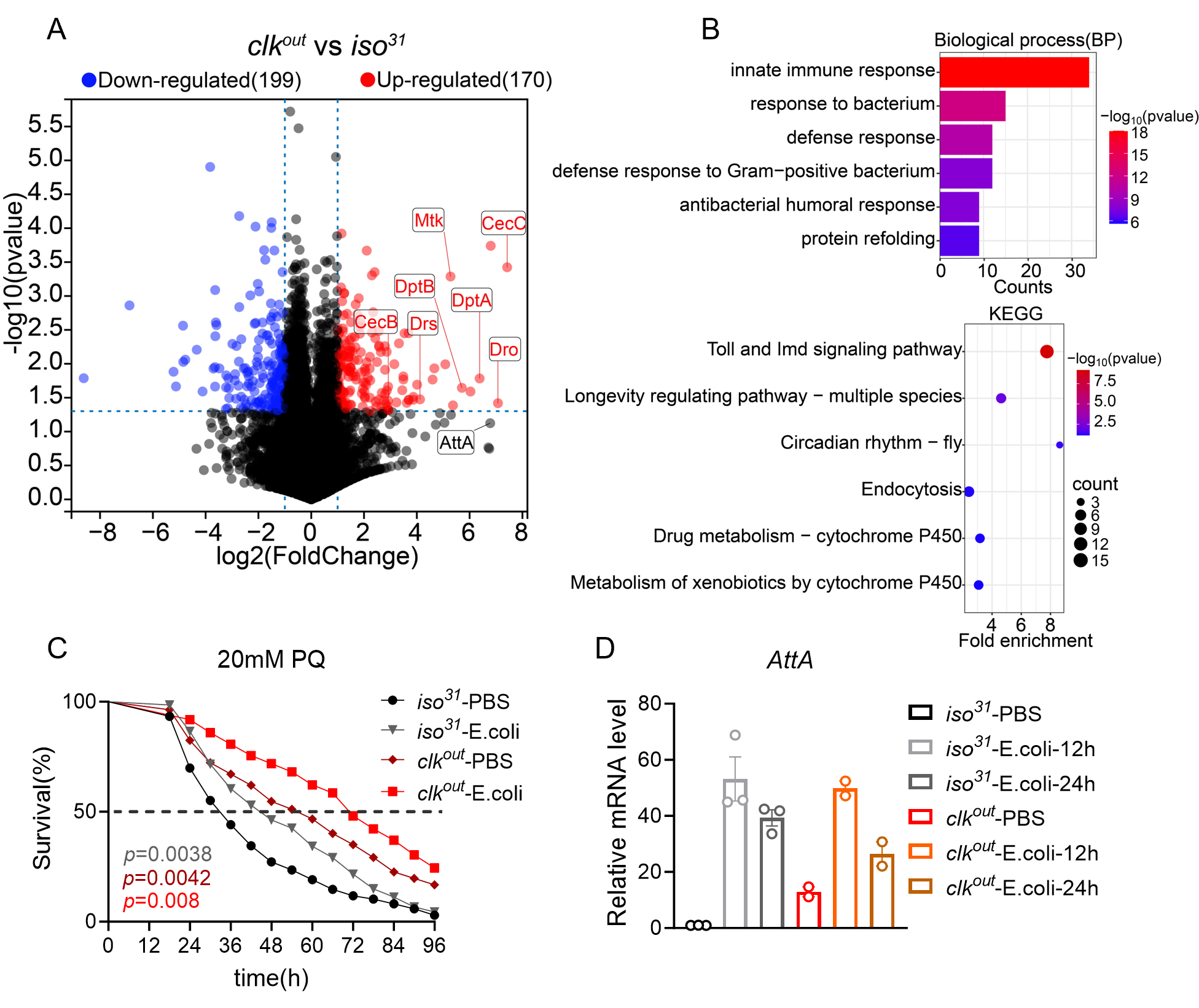


**Figure S2. CLK regulates AMP gene expression under oxidative and infectious stress.**

(A) Volcano plot of differentially expressed genes (DEGs) in *clk^out^* versus *iso^31^* flies at 24 hours following PQ treatment. Significantly upregulated (red) and downregulated (blue) genes are highlighted; non-significant genes are shown in gray. DEGs were defined by |fold change| > 2 and P < 0.05. Data represent three independent biological replicates.

(B) Enrichment analysis of DEGs at 24 h post-PQ. Top: Bar plot of significantly enriched immune- and stress-related pathways, ranked by –log₁₀(P-value). Bottom: Bubble plot visualization, where dot size indicates gene count per pathway and color scale reflects statistical significance.

(C) Survival analysis of flies subjected to combined oxidative and infectious stress. Flies were injected with heat-inactivated E. coli or PBS as control, followed by PQ treatment. *clk^out^* flies exhibited differential survival responses compared to *iso^31^* under both challenge conditions. n = 134–137 per group; statistical significance determined using log-rank test.

(D) Expression of AMP genes *AttA* in *iso^31^* and *clk^out^* flies at 12 and 24 hours post-injection with PBS or E. coli. Gene expression was normalized to PBS-injected *iso^31^* controls. Data are shown as mean ± SEM from three independent experiments.


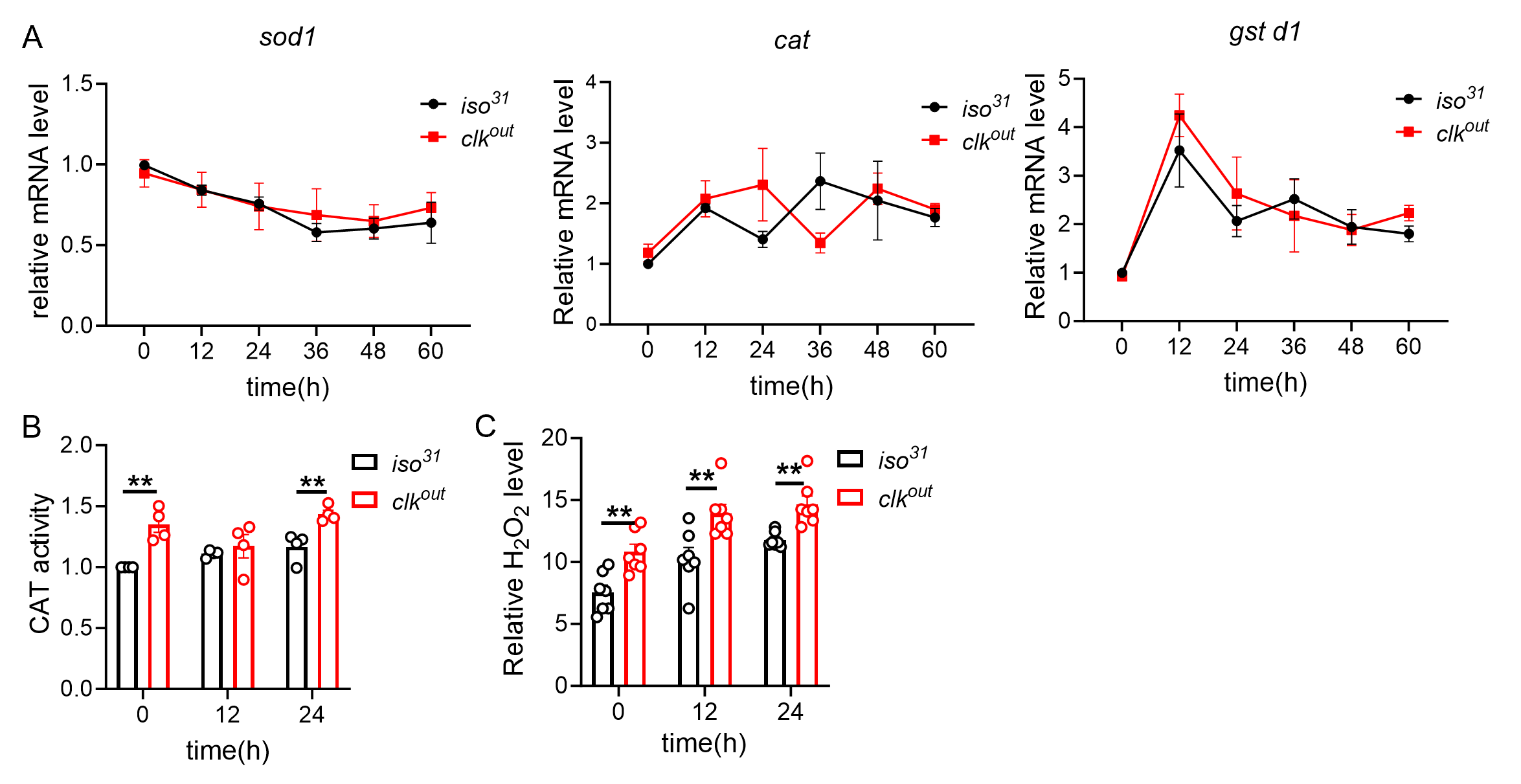


**Figure S3. *Clk* deficiency alters redox homeostasis and enhances antioxidant responses.**

(A) Temporal expression profiles of antioxidant-related genes (*sod1*, *cat*, and *gstd1*) in *iso^31^* and *clk^out^* flies over a 60-hour period. mRNA levels were normalized to 0 h expression in *iso^31^* controls. *clk^out^* flies exhibited consistently elevated expression of these oxidative stress-associated transcripts. Data are from three biological replicates.

(B) Catalase (CAT) enzymatic activity measured at indicated time points. *clk^out^* flies showed significantly increased CAT activity compared to *iso^31^*. Statistical significance determined using two-tailed unpaired t-test (**P < 0.01). Data are from four biological replicates.

(C) Quantification of hydrogen peroxide (H₂O₂) levels revealed heightened oxidative stress in *clk^out^* flies. Data represent mean ± SEM from three independent biological replicates. Statistical significance determined using two-tailed unpaired t-test (**P < 0.01).


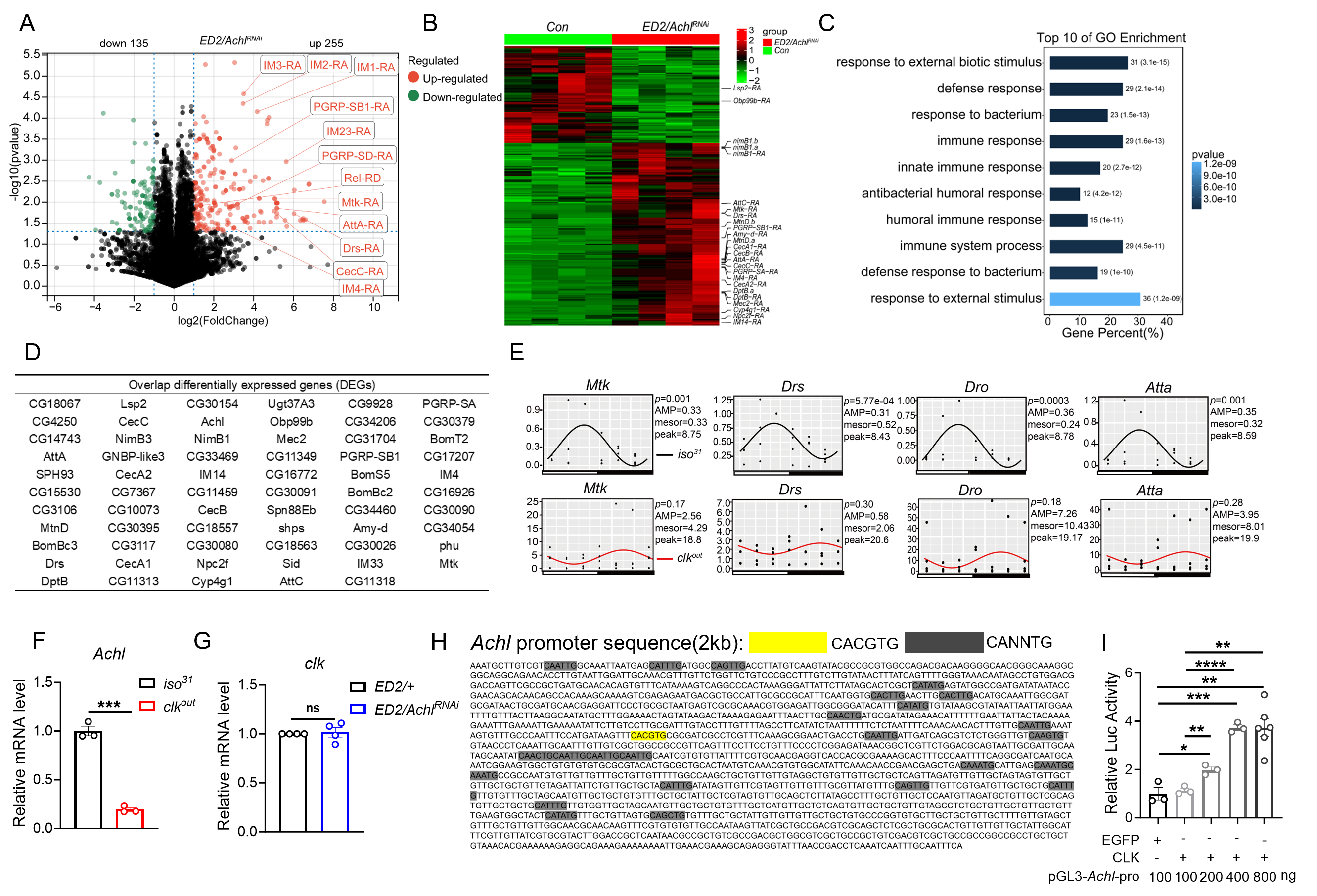


**Figure S4. CLK and ACHL coordinately regulate circadian expression of AMP genes in *Drosophila*.**

(A) Volcano plot showing DEGs in *Elav-GAL4*;; *UAS-dicer2/Achl^RNAi^* flies compared to controls. Upregulated (green) and downregulated (red) genes were identified using |fold change| > 2 and P < 0.05.

(B) Hierarchical clustering heatmap displaying global transcriptional changes induced by *Achl* knockdown.

(C) Gene Ontology (GO) enrichment analysis of DEGs reveals significant overrepresentation of categories such as “response to external biotic stimulus.” (A-C) Data were reanalyzed from reference. ^[37]^

(D) Overlap analysis between DEGs and predefined immune/stress-related gene sets highlights shared targets, including *Mtk*, *Drs*, *AttA*, and *Achl*.

(E) Circadian expression parameters of AMP genes (*Mtk*, *Drs*, *AttA*, *Dro*) assessed by Cosinor-circacompare in *clk^out^* and *iso^31^* flies. Alterations in amplitude, mesor, and phase indicate disrupted rhythmicity in *clk* mutants. Data are from four biological replicates.

(F) *Achl* transcript levels were markedly reduced in *clk^out^* flies, confirming positive regulation by CLK. ***P < 0.001 by two-tailed unpaired t-test. Data are from three biological replicates.

(G) *clk* mRNA expression was unchanged in *Elav-GAL4*;; *UAS-dicer2/Achl^RNAi^* flies, indicating that *Achl* does not regulate *clk* in a feedback manner. Data are from three biological replicates.

(H) Sequence alignment of the *Achl* promoter reveals conserved E-box motifs (CACGTG), indicative of CLK binding sites.

(I) Dual-luciferase reporter assays show that CLK activates the *Achl* promoter in a dose-dependent manner. Statistical analysis by one-way ANOVA with Tukey’s post hoc test: *P < 0.05, **P < 0.01, ***P < 0.001, ****P < 0.0001. Data represent mean ± SEM from at least three biological replicates.


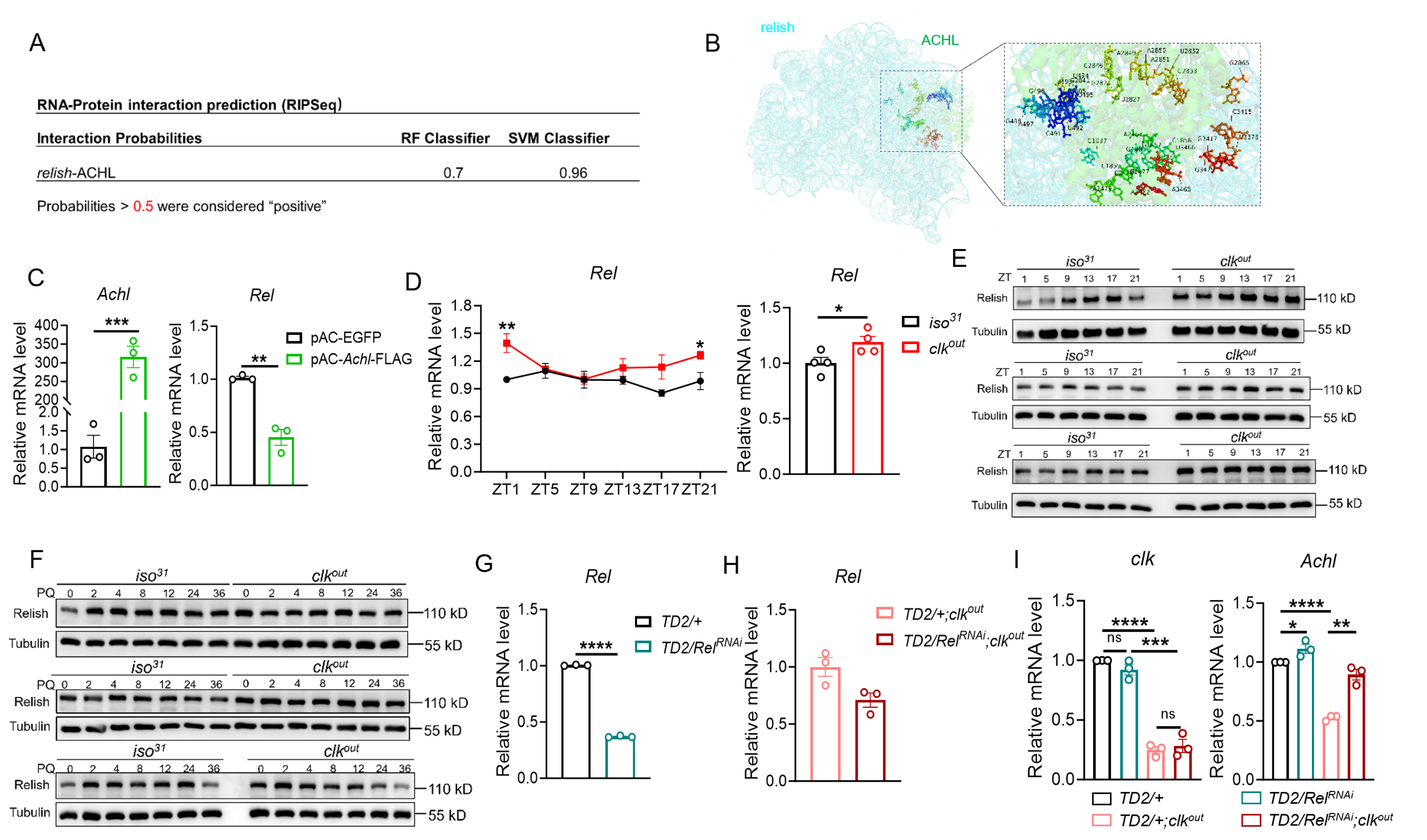


**Figure S5. Relish functions downstream of the CLK–ACHL axis to regulate oxidative stress responses in *Drosophila*.**

(A) RNA–protein interaction prediction using RIP-seq data combined with machine learning classifiers (Random Forest and Support Vector Machine) reveals high-probability interaction between ACHL and *Relish* mRNA (interaction threshold > 0.5).

(B) Structural prediction suggests potential interaction sites between ACHL and *Relish* mRNA, based on AlphaFold3 modeling. pTM=0.28, ipTM=0.26. The large dashed box (left) indicates the predicted interaction interface. The magnified view (right) highlights local RNA nucleotides that may participate in potential contacts, with different residue clusters colored separately (blue: G491–G497; green: A2467–C1855; yellow: A2849–U2853; orange: C3415–G3474; red: U3466–A3465).

(C) qRT-PCR analysis of *Achl* and *Relish* mRNA levels in S2 cells transfected with ACHL-FLAG or vector control (pAC-EGFP). **P < 0.01, ***P < 0.001 by two-tailed unpaired t-test. Data are from three biological replicates.

(D) Temporal expression profiling of *Relish* mRNA across six Zeitgeber time (ZT) points in *iso^31^* and *clk^out^* flies. *clk^out^* mutants exhibit consistently elevated *Relish* expression. *P < 0.05, **P < 0.01 by two-tailed unpaired t-test. Data are from four biological replicates.

(E) Western blot analysis of circadian Relish protein levels (ZT1–ZT21) in *iso^31^* and *clk^out^* flies. Tubulin serves as a loading control. Data are from three biological replicates.

(F) Relish protein expression under PQ-induced oxidative stress (0–36 h) is more sustained and elevated in *clk^out^* mutants than in *iso^31^* controls. Data are from three biological replicates.

(G–H) qPCR validation of *Relish* knockdown in *tim-GAL4*; *UAS-dicer2/Relish^RNAi^* flies in *iso^31^* (G) and *clk^out^* (H) backgrounds. ****P < 0.0001 by two-tailed unpaired t-test. Data are from three biological replicates.

(I) *clk* and *Achl* mRNA levels remain unchanged in *TD2* flies with or without *Relish* knockdown in both *iso^31^* and *clk^out^* backgrounds. Data represent mean ± SEM; *P < 0.05, **P < 0.01, ***P < 0.001, ****P < 0.0001 one-way ANOVA with Tukey’s post hoc test. Data are from three biological replicates.


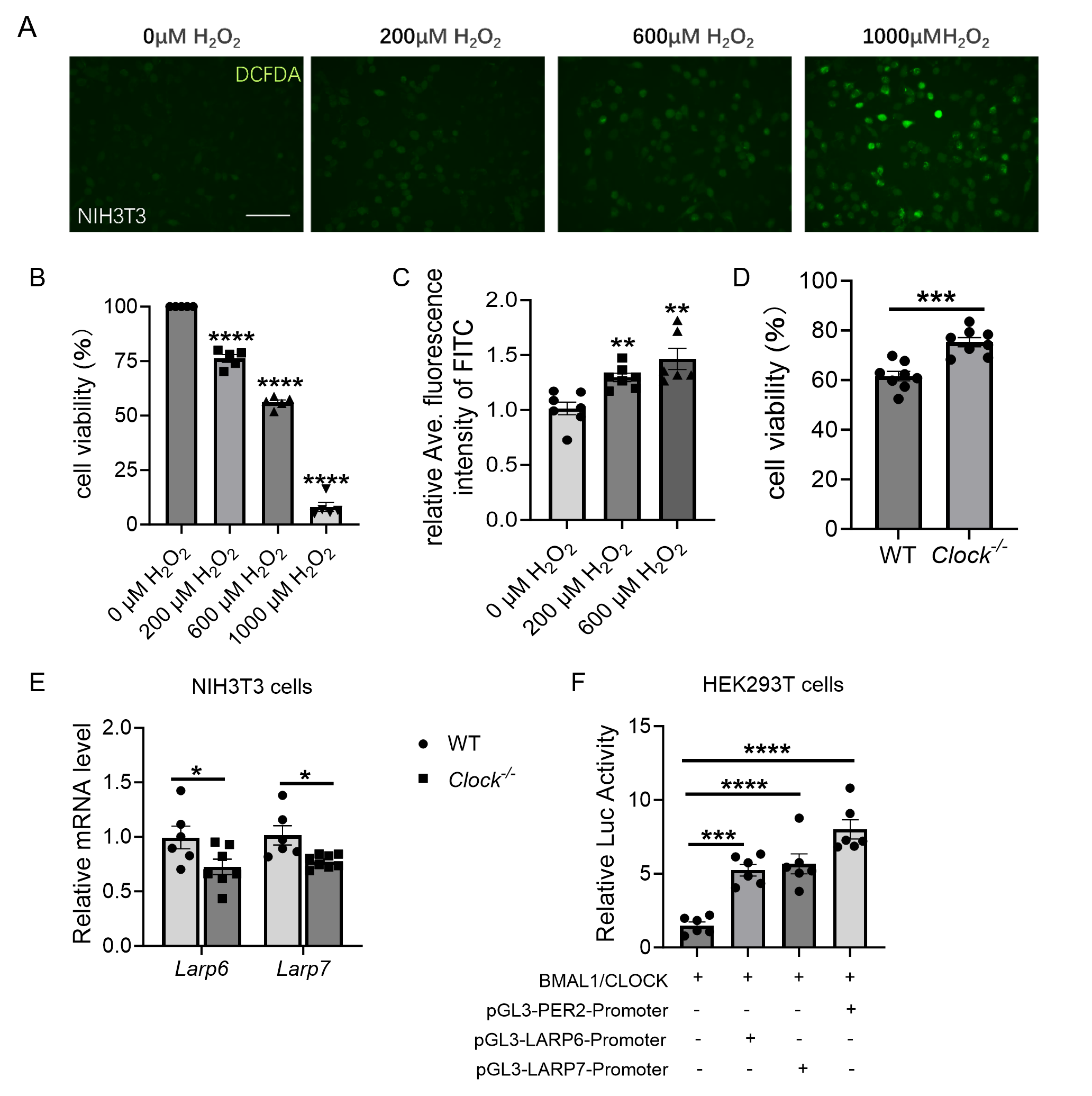


**Figure S6. Loss of *Clock* confers resistance to oxidative stress in NIH3T3 cells.**

(A) Representative DCFH-DA fluorescence images showing reactive oxygen species (ROS) levels in NIH3T3 cells treated with increasing concentrations of hydrogen peroxide (0, 200, 600, and 1000 μM) for 24 hours. Scale bar:50 μm.

(B) MTT-based cell viability assay of NIH3T3 cells after 24-hour exposure to H₂O₂. Cell viability declines significantly in a concentration-dependent manner. Data are from six biological replicates; ****P < 0.0001 (one-way ANOVA with Tukey’s post hoc test.).

(C) Quantification of FITC signal reveals a dose-dependent increase in intracellular ROS by flow cytometry. Data represent mean ± SEM from ≥5 independent experiments. **P < 0.01 (one-way ANOVA with Tukey’s post hoc test.).

(D) Comparative analysis of oxidative stress-induced cytotoxicity in wild-type (WT) and *Clock*-knockout (*Clock^-/-^*) NIH3T3 cells. *Clock^-/-^* cells exhibit significantly higher viability under 600 μM H₂O₂, indicating a specific role of CLOCK in promoting oxidative stress susceptibility. ***P < 0.001; (two-tailed unpaired t-test.). Data are from three biological replicates.

(E) Quantitative PCR analysis showing the relative mRNA levels of *Larp6* and *Larp7* in WT and *Clock^−/−^* NIH-3T3 cells. Data represent mean ± SEM from three independent experiments. Statistical significance was determined using a two-tailed unpaired t-test. *P < 0.05.

(F) Dual-luciferase reporter assay measuring the relative luciferase activity in HEK293T cells co-transfected with BMAL1/CLOCK and pGL3-based reporter constructs containing the proximal promoters of PER2, LARP6, or LARP7. Data are presented as mean ± SEM from three independent experiments. Statistical significance was determined using one-way ANOVA with Tukey’s post hoc test. ***P < 0.001, ****P < 0.0001.
